# 11β,20β-Epoxybriaranes from the Gorgonian Coral *Junceella fragilis* (Ellisellidae)

**DOI:** 10.3390/md18040183

**Published:** 2020-03-31

**Authors:** Tung-Pin Su, Tsu-Jen Kuo, San-Nan Yang, Gene-Hsiang Lee, Yen-Tung Lee, Yi-Chen Wang, Jih-Jung Chen, Zhi-Hong Wen, Tsong-Long Hwang, Ping-Jyun Sung

**Affiliations:** 1 Graduate Institute of Marine Biology, National Dong Hwa University, Pingtung 94450, Taiwan; g3xz84120@yahoo.com.tw; 2 National Museum of Marine Biology and Aquarium, Pingtung 94450, Taiwan; 3 Department of Stomatology, Kaohsiung Veterans General Hospital, Kaohsiung 81362, Taiwan; tsujenkuo@gmail.com; 4 Department of Marine Biotechnology and Resources, National Sun Yat-sen University, Kaohsiung 80424, Taiwan; wzh@mail.nsysu.edu.tw; 5 Department of Dental Technology, Shu-Zen Junior College of Medicine and Management, Kaohsiung 82144, Taiwan; 6 Department of Pediatrics, E-DA Hospital, School of Medicine, College of Medicine, I-SHOU University, Kaohsiung 82445, Taiwan; y520729@gmail.com; 7 Instrumentation Center, National Taiwan University, Taipei 10617, Taiwan; ghlee@ntu.edu.tw; 8 Division of Natural Products, Graduate Institute of Biomedical Sciences, College of Medicine, Chang Gung University, Taoyuan 33302, Taiwan; b9105008@stmail.cgu.edu.tw; 9 Department of Cosmetic Science, College of Human Ecology, Chang Gung University of Science and Technology, Taoyuan 33303, Taiwan; 10 Department of Chinese Medicine, MacKay Memorial Hospital, Taipei 10449, Taiwan; 11 Division of Cardiology, Department of Internal Medicine, Kaohsiung Armed Forces General Hospital, Kaohsiung 80284, Taiwan; cvyc.wang@gmail.com; 12 Faculty of Pharmacy, School of Pharmaceutical Sciences, National Yang-Ming University, Taipei 11221, Taiwan; chenjj@ym.edu.tw; 13 Research Center for Chinese Herbal Medicine, Research Center for Food and Cosmetic Safety, Graduate Institute of Healthy Industry Technology, College of Human Ecology, Chang Gung University of Science and Technology, Taoyuan 33303, Taiwan; 14 Graduate Institute of Natural Products, College of Medicine, Chang Gung University, Taoyuan 33302, Taiwan; 15 Chinese Herbal Medicine Research Team, Healthy Aging Research Center, Chang Gung University, Taoyuan 33302, Taiwan; 16 Department of Anaesthesiology, Chang Gung Memorial Hospital, Taoyuan 33305, Taiwan; 17 Chinese Medicine Research and Development Center, China Medical University Hospital, Taichung 40447, Taiwan; 18 Graduate Institute of Natural Products, Kaohsiung Medical University, Kaohsiung 80708, Taiwan

**Keywords:** *Junceella fragilis*, fragilide, briarane, juncenolide, X-ray, iNOS, COX-2

## Abstract

Two 11,20-epoxybriaranes, including a known compound, juncenolide K (**1**), as well as a new metabolite, fragilide X (**2**), have been isolated from gorgonian *Junceella fragilis* collected off the waters of Taiwan. The absolute configuration of juncenolide K (**1**) was determined by single-crystal X-ray diffraction analysis for the first time in this study and the structure, including the absolute configuration of briarane **2** was established on the basis of spectroscopic analysis and compared with that of model compound **1**. One aspect of the stereochemistry of the known compound **1** was revised. Briarane **2** was found to enhance the generation of inducible nitric oxide synthase (iNOS) and cyclooxygenase-2 (COX-2) release from RAW 264.7 cells.

## 1. Introduction

**Figure 1:**
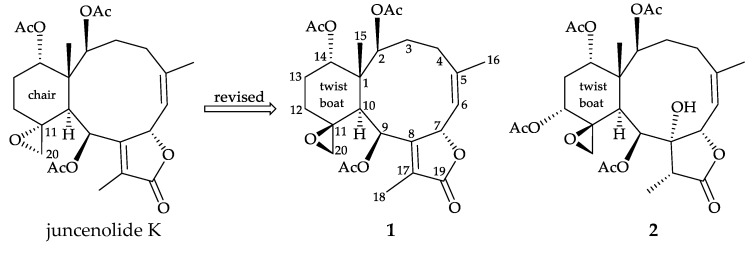
The structures of juncenolide K and its revised structure (**1**) and fragilide X (**2**).

Gorgonian corals of the genus *Junceella* (family Ellisellidae) [1,2,3] were proven to be the most important flagship species to produce 11,20-epoxybriarane diterpenoids, a chemical marker for the octocorals belonging to the family Ellisellidae [4,5] and the compounds of this type demonstrate a wide spectrum of biological properties, such as anti-inflammatory activity [6,7,8,9,10,11,12,13,14,15,16,17,18,19,20], immunomodulatory activity [21], insecticidal activity [22], cytotoxicity [23,24,25,26,27,28,29,30,31,32], anti-viral activity [6,33], anti-fouling activity [34,35,36,37], antifeedant [35], and anti-microbial activity [28,29,32,38,39,40]. From the specimens of *J. fragilis* (Ridley 1884) collected off the waters of Taiwan, an area with high biodiversity at the intersection of the Kuroshio current and the South China Sea surface current, we have isolated two briaranes, including a known compound juncenolide K (**1**) [13], along with a new briarane–fragilide X (**2**), featuring an 11,20-epoxy moiety in their structures (Figure 1). A pro-inflammatory assay was employed to assess the activity of these isolates on the release of inducible nitric oxide synthase (iNOS) and cyclooxygenase-2 (COX-2) from RAW 264.7 macrophage cells.

## 2. Results and Discussion

**Figure 2:**
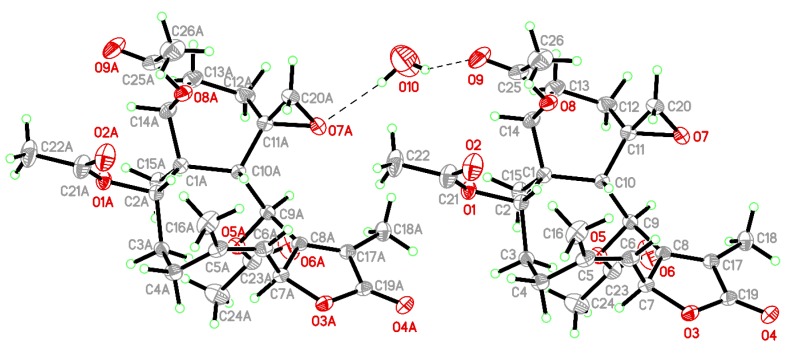
The Oak Ridge Thermal Ellipsoid Plot (ORTEP) of **1**.

**Figure 3:**
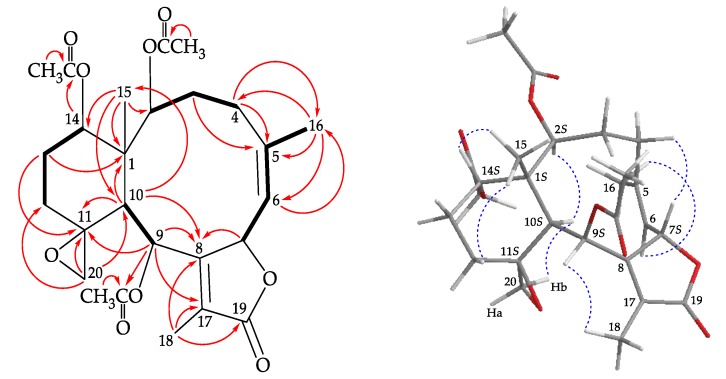
The COSY (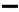) correlations, selective HMBC correlations (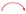), and selective protons with key NOESY (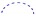) correlations of **1**.

Compound **1** was isolated as a colorless prism that showed a sodiated adduct ion [M + Na]^+^ at *m/z* 513.20949 in the (+)-high-resolution electrospray ionization mass spectrum (HRESIMS) analysis. The result revealed that **1** had a molecular formula of C_26_H_34_O_9_ (calculated for C_26_H_34_O_9_ + Na, 513.20950) (unsaturation degrees = 10). The NMR chemical shifts for **1** and its proton coupling data are identical to those reported for juncenolide K [13] (Table 1). Juncenolide K was initially assigned possessing an 11α,20α-epoxy configuration, and the cyclohexane ring was reported to exist with a chair conformation, but on the basis of our study of juncenolide K by a single-crystal X-ray diffraction analysis (Figure 2) and spectroscopic analysis (Table 1 and Figure 3) (Supplementary Materials, Figures S1–S14), it appears that the 11,20-epoxy group in **1** was found to be 11β,20β-oriented and **1** possesses a cyclohexane ring in twist-boat form. The X-ray structure shows the twist-boat conformation of the cyclohexane ring in **1** and the Oak Ridge Thermal Ellipsoid Plot (ORTEP) diagram (Figure 2) showed that the absolute configurations of the stereogenic centers of **1** are 1*S*,2*S*,7*S*,9*S*,10*S*,11*S* and 14*S* (Flack parameter x = 0.07(5)).

Fragilide X (**2**) was isolated as an amorphous powder and displayed a sodiated adduct ion [M + Na]^+^ at *m/z* 589.22576 in the (+)-HRESIMS, indicating a molecular formula C_28_H_38_O_12_ (calculated for C_28_H_38_O_12_ + Na, 589.22555) (unsaturation degrees = 10). Absorption peaks at 3333 cm^–1^, 1773 cm^–1^, and 1742 cm^–1^ in the IR spectrum indicate hydroxy, γ-lactone, and ester groups, respectively. Analysis of the ^1^H, ^13^C NMR, and distortionless enhancement by polarization transfer (DEPT) spectra, together with the molecular formula, suggested that there must be an exchangeable proton. The ^13^C NMR spectrum (Table 2), in combination with DEPT, HSQC, and HMBC spectra, revealed the presence of five esters including four acetoxy groups (δ_C_ 21.6, 21.0, 20.9, 20.7, 4 × CH_3_; δ_C_ 170.6, 169.8, 169.4, 169.2, 4 × C) and a lactone moiety (δ_C_ 176.2), and a trisubstituted olefin (δ_C_ 143.8, C-5; 120.8, CH-6). Based on the ^13^C NMR data and numbers of unsaturation, **2** was established as a tetracyclic diterpenoid. The presence of an exocyclic epoxy group was confirmed from the signals of an oxygenated quaternary carbon at δ_C_ 62.3 (C-11) and an oxymethylene at δ_C_ 53.9 (CH_2_-20). The chemical shifts of oxymethylene protons at δ_H_ 3.20 (1H, d, *J* = 4.4 Hz, H-20a) and 2.90 (1H, d, *J* = 4.4 Hz, H-20b) further supported the presence of this group. Moreover, a methyl singlet, two methyl doublets (including a vinyl methyl), three pairs of sp^3^ methylene protons, two sp^3^ methine protons, five oxymethine protons, an sp^2^ methine proton, four acetate methyls, and a hydroxy proton were observed in the ^1^H NMR spectrum (Table 2).

**Figure 4:**
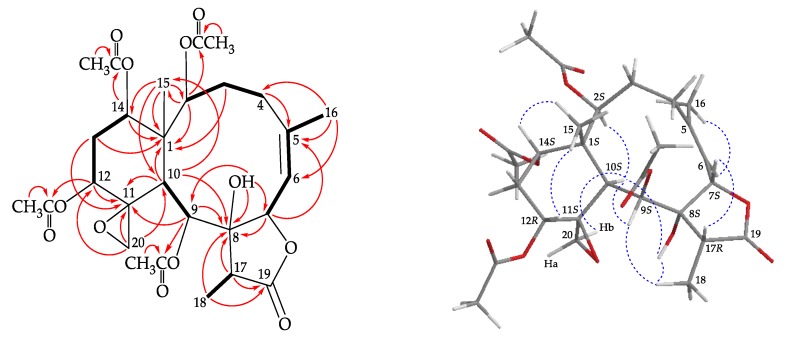
The COSY (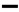) correlations, selective HMBC correlations (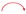), and selective protons with key NOESY (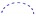) correlations of **2**.

The ^1^H NMR coupling information in the correlation spectroscopy analysis enabled the determination of five different spin systems, H-2/H_2_-3/H_2_-4, H-6/H-7, H-9/H-10, H-12/H_2_-13/H-14, and H-17/H_3_-18, which were assembled with the assistance of an HMBC experiment (Figure 4). The HMBC correlations between protons and quaternary carbons, such as H-2, H-3β, H-10, H-13α, H-14, H_3_-15 to C-1; H_2_-4, H-7, H_3_-16 to C-5; H-7, H-9, H-10, H-17, H_3_-18 to C-8; H-9, H-10, H-12, H_2_-20, H_2_-13 to C-11; and H-17, H_3_-18 to C-19, respectively, permitted elucidation of the carbon skeleton of **2**. A methyl at C-5 was confirmed by the HMBC correlations between H_3_-16 to C-4, C-5, and C-6; and further confirmed by an allylic coupling between H-6/H_3_-16 (*J* = 1.2 Hz). The methyl group Me-15 on C-1 was substantiated by the HMBC correlations from H_3_-15 to C-1, C-2, C-10, C-14; and H-2, H-10 to C-15, respectively. The epoxy group at C-11/20 was confirmed by the HMBC correlations between H_2_-20 to C-10, C-11, C-12. The hydroxy group at C-8 was deduced from the HMBC correlations of a hydroxy proton at δ_H_ 4.57 to C-7, C-8, and C-9. Moreover, HMBC correlations from the oxymethine protons at δ_H_ 4.74 (H-2), 5.67 (H-9), 5.40 (H-12), and 4.85 (H-14) to the acetate carbonyls at δ_C_ 170.6, 169.2, 169.4, and 169.8, placed the acetoxy groups on C-2, C-9, C-12, and C-14, respectively. 

The stereochemistry of **2** was determined by NOE correlations observed in a NOESY experiment (Figure 4) and possible biogenetic considerations. The NOE correlations of H-10/H-2, H-10/OH-8, H-10/H-9, and H-10/H-20b indicated that these protons are situated on the same face of the structure and were assigned as the α protons since the C-15 methyl is the β-substituent at C-1. Meanwhile, correlations of H_3_-15/H-12 and H_3_-15/H-14 indicated that H-12 and H-14 were β-oriented, and the cyclohexane ring may exhibit a twist-boat conformation. The NOESY spectrum showed a correlation from H-6 to H_3_-16, revealing the *Z* geometry of the C-5/6 double bond. H_3_-18 exhibited correlations to OH-8 and H-9, suggesting the α-orientation of Me-18 at C-17. H-7 displayed a correlation with H-17, which confirmed that these two protons were β-oriented at C-7 and C-17, respectively. As briarane **2** was isolated along with **1** from the same organism, it is reasonable on biogenetic grounds to assume that **2** possessed the same absolute configuration as that of **1**. Therefore, the configurations of the stereogenic carbons of **2** should be assigned as 1*S*,2*S*,7*S*,8*S*,9*S*,10*S*,11*S*,12*R*,14*S*, and 17*R* (Supplementary Materials, Figures S15–S29).

**Figure 5:**
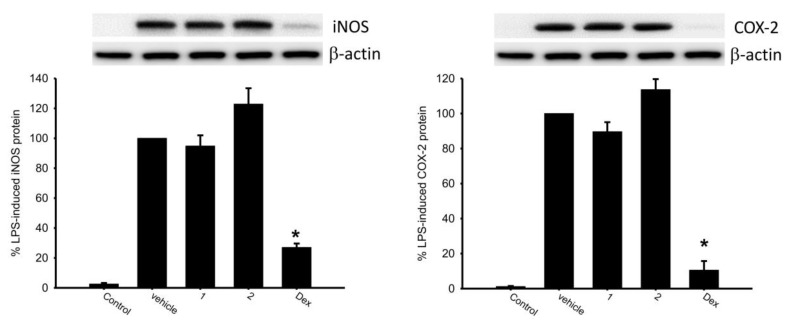
Western blotting showed that briarane **2** enhanced the expression of iNOS and COX-2. Data were normalized to the cells treated with LPS only, and cells treated with dexamethasone (Dex) (10 μM) were used as a positive control. Data are presented as the mean ± SEM (*n* = 3). * Significantly different from cells treated with LPS (*p* < 0.05).

The effects of briaranes **1** and **2** on the release of iNOS and COX-2 from lipopolysaccharide (LPS)-stimulated RAW 264.7 macrophage cells were assessed (Table 3 and Figure 5). It is interesting to note that **2** at 10 μM enhanced the release of iNOS and COX-2 to 122.87% and 113.65%, respectively, as compared to results of the cells stimulated with LPS only.

## 3. Materials and Methods

### 3.1. General Experimental Procedures

NMR spectra were recorded on a 400 MHz Jeol NMR (model ECZ 400S, Tokyo, Japan) spectrometer using the residual CHCl_3_ signal (δ_H_ 7.26 ppm) and CDCl_3_ (δ_C_ 77.1 ppm) as internal references for ^1^H and ^13^C NMR, respectively. ESIMS and HRESIMS were obtained from a Bruker mass spectrometer with 7 Tesla magnets (model: SolariX FTMS system, Bremen, Germany). Column chromatography, HPLC, IR spectra, and optical rotation values were performed according to our earlier research [19].

### 3.2. Animal Material

The specimens coral *J. fragilis* were collected in July 2019 by hand, using self-contained underwater breathing apparatus (SCUBA) off the coast of Orchid Island (Lanyu Island), Taiwan. The samples were stored in a −20 °C freezer until extraction. A voucher specimen was deposited in the National Museum of Marine Biology and Aquarium (NMMBA) (voucher no.: NMMBA-TW-GC-2019-017). This organism was identified by comparison with previous descriptions [1,2,3].

### 3.3. Extraction and Isolation

Sliced bodies (wet/dry weight = 1125 g/588 g) of the coral specimen were prepared and extracted with a mixture of methanol (MeOH) and dichloromethane (CH_2_Cl_2_) (1:1) to give a crude extract (29.0 g) which was partitioned between ethyl acetate (EtOAc) and H_2_O. The EtOAc extract (17.0 g) was then applied to a silica gel column chromatograph (C.C.) (500 g) and eluted with gradients of hexanes/acetone (stepwise from 50:1 (3000 mL)-30:1 (3000 mL)-20:1 (3000 mL)-10:1 (3000 mL)-5:1 (3000 mL)-4:1 (3000 mL)-3:1 (3000 mL)-2:1 (3000 mL)-1:1 (3000 mL)-1:2 (3000 mL)) to furnish fractions A−J. Fraction F (913.9 mg) was separated on silica gel C.C. and eluted with gradients of hexanes/acetone (stepwise from 20:1 (2400 mL)-15:1 (2400 mL)-10:1 (2400 mL)-8:1 (2400 mL)-6:1 (2400 mL)-4:1 (2400 mL)-2:1 (2400 mL)-1:1 (2400 mL) to furnish fractions F1−F8. Fraction F5 was further separated by silica gel C.C. with a mixture of hexanes/acetone (10:1 to 1:1, stepwise) to afford fractions F5A−F5F. Afterward, fraction F5C was separated by normal-phase HPLC (NP-HPLC) using a mixture of CH_2_Cl_2_ and acetone (10:1) to yield fractions F5C1−F5C4. Fraction F5C2 was purified by NP-HPLC using a mixture of *n*-hexane and EtOAc (2:1; at a flow rate = 2.0 mL/min) to afford **1** (32.4 mg). Fraction G was applied to a silica gel C.C. and eluted with a mixture of hexanes/acetone (3:1) to furnish fractions G1−G6. Fraction G4 was separated by silica gel C.C. using a mixture of CH_2_Cl_2_ and acetone (20:1) to afford fractions G4A− G4F. Fraction G4E was separated by NP-HPLC using a mixture of *n*-hexane/EtOAc/acetone (5:2:1) to yield fractions G4E1−G4E5. Fraction G4E4 was purified by NP-HPLC using a mixture of CH_2_Cl_2_ and acetone (10:1) to afford fractions G4E4A−G4E4C. Fraction G4E4A was separated by reverse-phase HPLC (RP-HPLC) using a mixture of acetonitrile and H_2_O (55:45; at a flow rate = 4.0 mL/min) to obtain **2** (0.7 mg).

Juncenolide K (**1**): Colorless crystals; ${\left[\alpha \right]}_{D}^{26}$ −90 (*c* 1.62, CHCl_3_) (ref. [13] ${\left[\alpha \right]}_{D}$ −85 (*c* 0.2, CH_2_Cl_2_)); IR (ATR) ν_max_ 2926, 1746, 1728, 1372, 1251, 1216, 759 cm^−1^; ^1^H (400 MHz, CDCl_3_) and ^13^C (100 MHz, CDCl_3_) NMR data, see Table 1; ESIMS: *m/z* 513 [M + Na]^+^; HRESIMS: *m/z* 513.20949 (calcd. for C_26_H_34_O_9_ + Na, 513.20950).

Fragilide X (**2**): Amorphous powder; ${\left[\alpha \right]}_{D}^{25}$ +232 (*c* 0.23, CHCl_3_); IR (KBr) ν_max_ 3333, 2942, 1773, 1742, 1374, 1219, 756 cm^−1^; ^1^H (400 MHz, CDCl_3_) and ^13^C (100 MHz, CDCl_3_) NMR data, see Table 2; ESIMS: *m/z* 589 [M + Na]^+^; HRESIMS: *m/z* 589.22576 (calcd. for C_28_H_38_O_12_ + Na, 589.22555).

### 3.4. Single-Crystal X-ray Crystallography of Juncenolide K (**1**)

Suitable colorless prisms of **1** were obtained from a solution of MeOH and petroleum ether. The crystal (0.255 × 0.233 × 0.114 mm^3^) belongs to the orthorhombic system, space group *P*2_1_2_1_2_1_ (#19), with *a* = 9.8842(2) Å, *b* = 15.5702(2) Å, *c* = 17.0502(3) Å, *V* = 2624.01(8) Å^3^, *Z* = 4, *D*_calcd_ = 1.264 Mg/m^3^, *λ* (Cu Kα) = 1.54178 Å. Intensity data were measured on a Bruker D8 Venture diffractometer up to *θ*_max_ of 75.0°. All 12,468 reflections were collected. The structure was solved by direct methods and refined by a full-matrix least-squares procedure [41,42]. The refined structural model converged to a final R1 (the R-value, is the agreement between the calculated and observed models) = 0.0396; wR2 (wR2 is similar to R1, but refers to squared F-values) = 0.1090 for 5385 observed reflections [*I* > 2σ(*I*)] and 335 variable parameters. The absolute configuration was determined by the Flack parameter x = 0.07(5) [43,44]. Crystallographic data for the structure of juncenolide K (**1**) were deposited with the Cambridge Crystallographic Data Center (CCDC) as supplementary publication number CCDC 1973681 [45]. 

### 3.5. In Vitro Inflammatory Assay

Murine RAW 264.7 macrophages were obtained from the American Type Culture Collection (ATCC; No. TIB-71). Inflammation in macrophages was induced by incubating them for 16 h in a medium containing only LPS (0.01 μg/mL) without compounds. For the anti-inflammatory activity assay, compounds (10 μM) were added to the cells 5 min before LPS challenge. The cells were then washed with ice cold phosphate-buffered saline (PBS), lysed in ice-cold lysis buffer (50 mM Tris, pH 7.5, 150 mM NaCl, 1% Triton X-100, 100 μg/mL phenylmethylsulfonyl fluoride, 1 μg/mL aprotinin), and then centrifuged at 20,000× *g* for 30 min at 4 °C. The supernatant was decanted from the pellet and retained for Western blot analysis of pro-inflammation inducible nitric oxide synthase (iNOS) and cyclooxygenase-2 (COX-2) protein expression. Protein concentrations were determined using the detergent compatible (DC) protein assay kit (Bio-Rad, Hercules, CA, USA). Western blotting was performed according to the method described in a previous study [46]. An equal volume of sample buffer (2% 2-mercaptoethanol, 2% sodium dodecyl sulfate (SDS), 0.1% bromophenol blue, 10% glycerol, and 50 mM Tris-HCl (pH 7.2)) was added to the samples, and the protein lysates were loaded onto a 10% SDS-polyacrylamide gel. Electrophoresis was carried out at 150 V for 90 min. After electrophoresis, gels were transferred overnight at 4 °C in transfer buffer (380 mM glycine, 50 mM Tris-HCl, 1% SDS and 20% methanol) onto a polyvinylidene difluoride membrane (PVDF; Immobilon-P, Millipore Corp. (0.45 μm pore size)). The PVDF membrane was first blocked with 5% non-fat dry milk in Tris-buffered saline containing 0.1% Tween (TTBS; 20 mM Tris-HCl, 0.1% Tween 20, and 137 mM NaCl (pH 7.4)) and incubated overnight at 4 °C with the primary antibodies for iNOS, COX-2, and β-actin proteins. Anti-iNOS and anti-COX-2 antibodies were purchased from Cayman Chemical Company (Ann Arbor, MI, USA). A horseradish peroxidase-conjugated secondary antibody was used for detection. It was obtained from Jackson ImmunoResearch Laboratories (West Grove, PA, USA). The bound antibodies were detected by chemiluminescence (Millipore Corp.). The images were obtained using the UVP BioChemi Imaging System, and the LabWorks 4.0 software (UVP, Upland, CA, USA) was used to quantify the relative densities.

## 4. Conclusions

*J. fragilis* has been demonstrated to have a wide structural diversity of briarane-type diterpenoids that possess various potential bioactivities. In our continued study on *J. fragilis*, a previously unreported 11,20-epoxybriarane, fragilide X (**2**), along with a known briarane, juncenolide K (**1**) were isolated. Revision of the structure and absolute configuration of juncenolide K (**1**) was confirmed by a single-crystal X-ray diffraction analysis. In the present study, a pro-inflammatory assay was employed to assess the activity of isolates, and fragilide X (**2**) was found to enhance the release of iNOS and COX-2, respectively.

## Figures and Tables

**Table 1:** ^1^H and ^13^C NMR (CDCl_3_) data for juncenolide K and briarane **1**.

	Juncenolide K ^a^		1	
Position	δ_H_ (*J* in Hz) ^b^	δ_C_, ^c^ type	δ_H_ (*J* in Hz) ^d^	δ_C_, ^e^ type
1		46.0, C		46.0, C
2	4.74 br s	74.1, CH	4.72 br s	74.1, CH
3	2.53–2.59 m; 1.74–1.80 m	31.3, CH_2_	2.56 m; 1.74 m	31.3, CH_2_
4	2.60–2.66 m; 2.20–2.26 m	29.1, CH_2_	2.60 m; 2.22 m	29.1, CH_2_
5		143.0, C		143.0, C
6	5.01 d (8.5)	124.7, CH	4.99 d (8.4)	124.8, CH
7	5.51 d (8.5)	77.2, CH	5.50 d (8.4)	77.1, CH
8		155.8, C		155.8, C
9	6.54 d (7.0)	66.5, CH	6.52 d (7.2)	66.6, CH
10	2.63–2.68 m	40.5, CH	2.64 br d (7.2)	40.5, CH
11		59.7, C		59.7, C
12	2.30–2.36 m; 1.10–1.16 m	22.9, CH_2_	2.31 m; 1.12 m	22.9, CH_2_
13	2.11–2.17 m; 1.80–1.86 m	23.8, CH_2_	2.10 m; 1.82 m	23.8, CH_2_
14	4.80 d (3.5)	73.9, CH	4.79 d (3.6)	73.9, CH
15	1.16 s	15.8, CH_3_	1.15 s	15.9, CH_3_
16	2.01 s	27.0, CH_3_	1.99 s	27.0, CH_3_
17		127.5, C		127.4, C
18	2.01 s	9.3, CH_3_	1.99 s	9.3, CH_3_
19		173.6, C		173.6, C
20a/b	2.60–2.66 m; 2.46–2.52 m	58.2, CH_2_	2.62 br s; 2.48 br s	58.2, CH_2_
OAc-2		170.7, C		170.7, C
	2.01 s	21.0, CH_3_	2.00 s	21.0, CH_3_
OAc-9		168.9, C		168.9, C
	2.12 s	21.6, CH_3_	2.11 s	21.7, CH_3_
OAc-14		169.8, C		169.8, C
	1.96 s	20.9, CH_3_	1.95 s	20.9, CH_3_

^a^ Data were reported by Wang et al., see ref. [13]. ^b^ 500 MHz, ^c^ 125 MHz, ^d^ 400 MHz, ^e^ 100 MHz.

**Table 2:** ^1^H and ^13^C NMR (CDCl_3_) data for **2**.

Position	δ_H_^a^ (*J* in Hz)	δ_C_, ^b^ type
1		46.8, C
2	4.74 d (4.8)	74.4, CH
3α/β	1.67 m; 2.43 ddd (16.0, 16.0, 4.4)	32.1, CH_2_
4α/β	2.52 br d (16.0); 2.07 m	28.7, CH_2_
5		143.8, C
6	5.61 br d (10.0)	120.8, CH
7	5.14 dd (10.0, 1.2)	77.7, CH
8		80.2, C
9	5.67 d (5.6)	67.3, CH
10	2.58 d (5.6)	39.9, CH
11		62.3, C
12	5.40 dd (8.4, 8.4)	62.0, CH
13α/β	1.58 m; 2.70 m	32.6, CH_2_
14	4.85 d (4.4)	73.6, CH
15	1.16 s	15.3, CH_3_
16	2.03 d (1.2)	28.1, CH_3_
17	2.35 q (7.2)	42.3, CH
18	1.15 d (7.2)	6.7, CH_3_
19		176.2, C
20a/b	3.20 d (4.4); 2.90 d (4.4)	53.9, CH_2_
OH-8	4.57 d (1.2)	
OAc-2		170.6, C
	2.01 s	21.0, CH_3_^c^
OAc-9		169.2, C
	2.22 s	21.6, CH_3_
OAc-12		169.4, C
	1.98 s	20.7, CH_3_^c^
OAc-14		169.8, C
	2.02 s	20.9, CH_3_^c^

^a^ 400 MHz, ^b^ 100 MHz, ^c^ Data exchangeable.

**Table 3:** Effects of briaranes **1** and **2** on lipopolysaccharide (LPS)-induced pro-inflammatory iNOS and COX-2 protein expression in macrophages.

Compound	iNOS		COX-2		β-Actin
	Expression (% of LPS)				
Control	2.59 ± 0.65		1.14 ± 0.34		100.15 ± 7.70
LPS	100.00 ± 0.00		100.00 ± 0.00		100.00 ± 0.00
**1**	94.81 ± 7.11		89.59 ± 5.45		101.09 ± 1.91
**2**	122.87 ± 10.53		113.65 ± 6.00		99.50 ± 1.64
Dexamethasone	26.99 ± 2.66		10.52 ± 5.23		99.02 ± 1.53

Data were normalized to those of cells treated with LPS alone, and cells treated with dexamethasone were used as a positive control. Data are expressed as the mean ± standard error of the mean (SEM) (*n* = 3).

## Supplementary Materials

Supplementary Materials are available online at https://www.mdpi.com/1660-3397/18/4/183/s1. HRESIMS, IR, 1D (^1^H, ^13^C NMR and DEPT spectra), and 2D (HSQC, HMBC, COSY, and NOESY) NMR spectra of juncenolide K (**1**) and fragilide X (**2**), and X-ray Crystallography of **1**.
