# Supplementary material for: 11β,20β-Epoxybriaranes from the Gorgonian Coral Junceella fragilis (Ellisellidae)
Source: Mar Drugs. 2020 Mar 31;18(4):183. doi: 10.3390/md18040183 (PMC7231240; doi:10.3390/md18040183)
Supplement: Supplementary file 1 [file marinedrugs-18-00183-s001.pdf]

S1. HRESIMS spectrum of compound **1** 2
S2. IR spectrum of compound **1** 2
S3. ^{1}H NMR spectrum (400 MHz) of compound **1** in $CDCl_3$ 3
S4. ^{13}C NMR spectrum (100 MHz) of compound **1** in $CDCl_3$ 3
S5. DEPT spectrum of compound **1** in $CDCl_3$ 4
S6. HSQC spectrum of compound **1** in $CDCl_3$ 4
S7. HSQC spectrum of compound **1** in $CDCl_3$ 5
S8. HSQC spectrum of compound **1** in $CDCl_3$ 5
S9. HMBC spectrum of compound **1** in $CDCl_3$ 6
S10. HMBC spectrum of compound **1** in $CDCl_3$ 6
S11. HMBC spectrum of compound **1** in $CDCl_3$ 7
S12. ^{1}H-^{1}H COSY spectrum of compound **1** in $CDCl_3$ 7
S13. NOESY spectrum of compound **1** in $CDCl_3$ 8
S14. Single-crystal X-ray Crystallography of compound **1** 9
S15. HRESIMS spectrum of compound **2** 20
S16. IR spectrum of compound **2** 20
S17. ^{1}H NMR spectrum (400 MHz) of compound **2** in $CDCl_3$ 21
S18. ^{13}C NMR spectrum (100 MHz) of compound **2** in $CDCl_3$ 21
S19. DEPT spectrum of compound **2** in $CDCl_3$ 22
S20. HSQC spectrum of compound **2** in $CDCl_3$ 22
S21. HSQC spectrum of compound **2** in $CDCl_3$ 23
S22. HSQC spectrum of compound **2** in $CDCl_3$ 23
S23. HMBC spectrum of compound **2** in $CDCl_3$ 24
S24. HMBC spectrum of compound **2** in $CDCl_3$ 24
S25. HMBC spectrum of compound **2** in $CDCl_3$ 25
S26. HMBC spectrum of compound **2** in $CDCl_3$ 25
S27. HMBC spectrum of compound **2** in $CDCl_3$ 26
S28. ^{1}H-^{1}H COSY spectrum of compound **2** in $CDCl_3$ 26
S29. NOESY spectrum of compound **2** in $CDCl_3$ 27

Mass Spectrum SmartFormula Report

Analysis Info

Analysis Name	D:\Data\a2\LU65323_000003.d	1/7/2020 3:41:35 PM
Method	broadband first signal	Operator: YU HSIAO-CHING
Sample Name	LU-6-5-3-2-3	Instrument: BRUKER FT-MS solariX
Comment	ESI Positive	

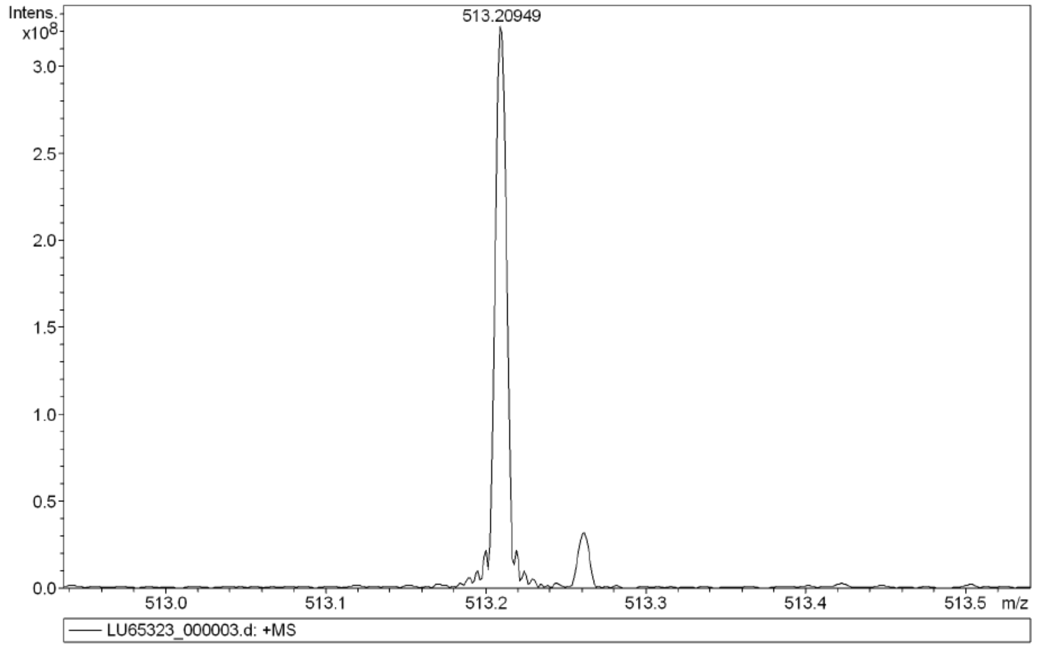

Meas. m/z	#	Formula	Score	m/z	err [mDa]	err [ppm]	mSigma	rdb	e⁻ Conf	N-Rule
513.20949	1	C 26 H 34 Na O 9	100.00	513.20950	0.02	0.03	8.1	9.5	even	ok

S1. HRESIMS spectrum of compound **1**

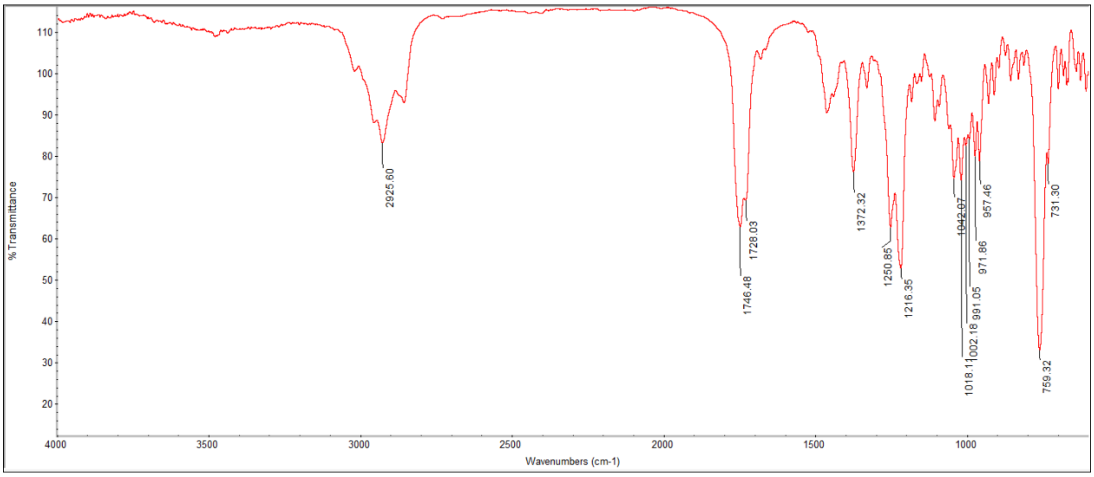

S2. IR spectrum of compound **1**

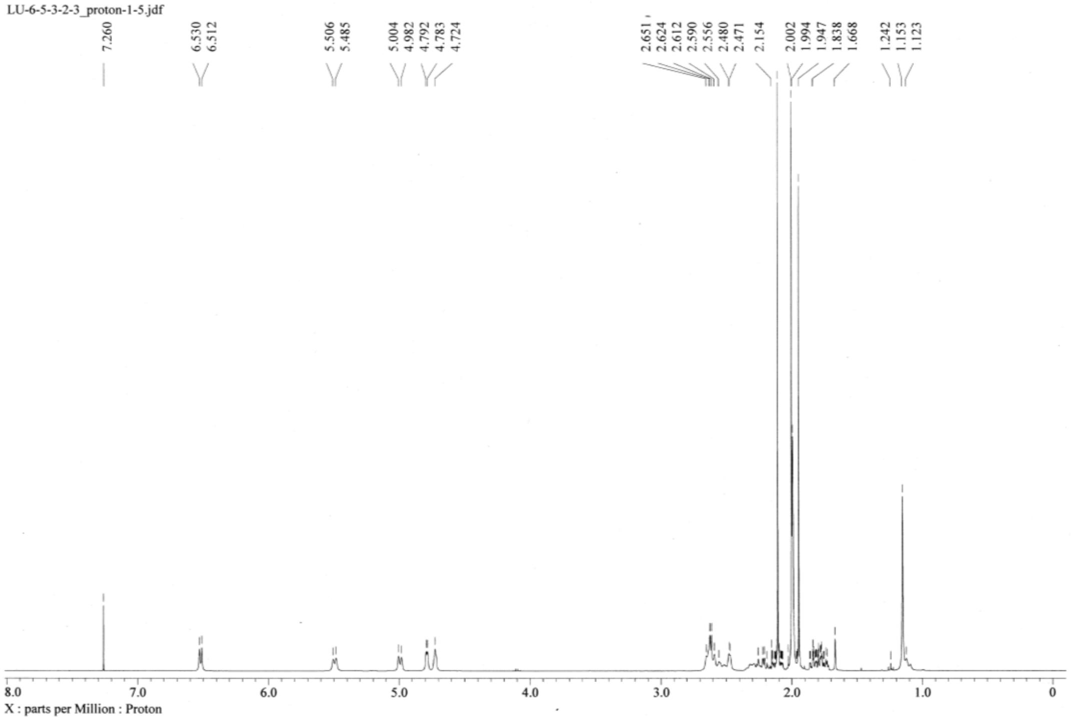

S3. ^{1}H NMR spectrum (400 MHz) of compound **1** in $CDCl_3$

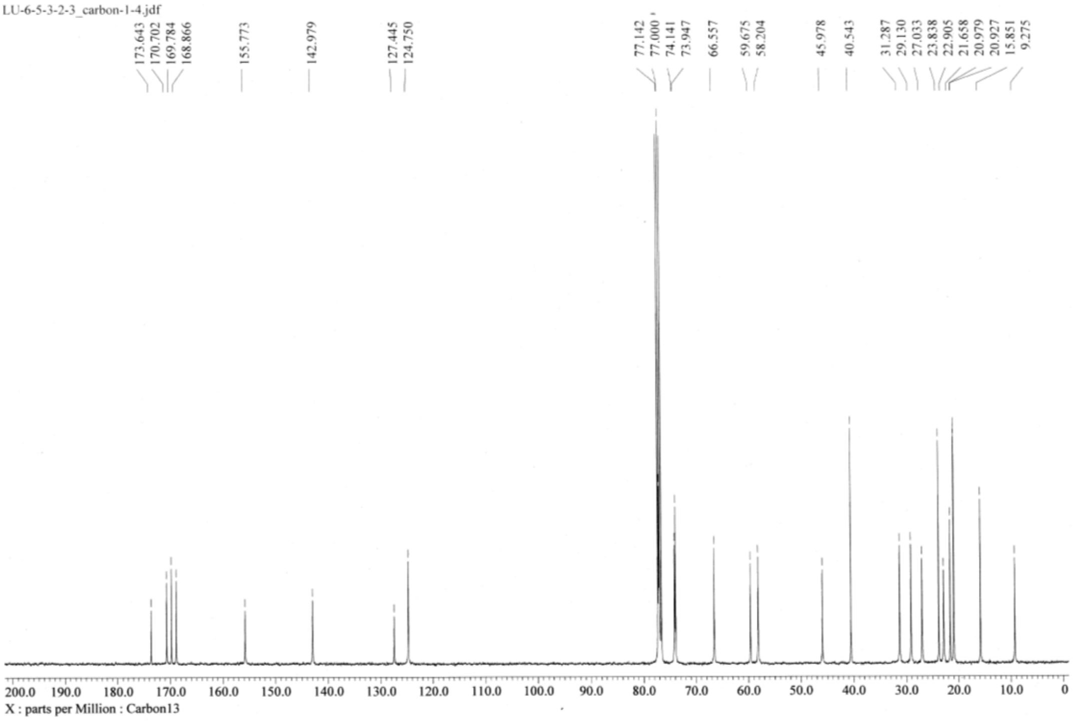

S4. ^{13}C NMR spectrum (100 MHz) of compound **1** in $CDCl_3$

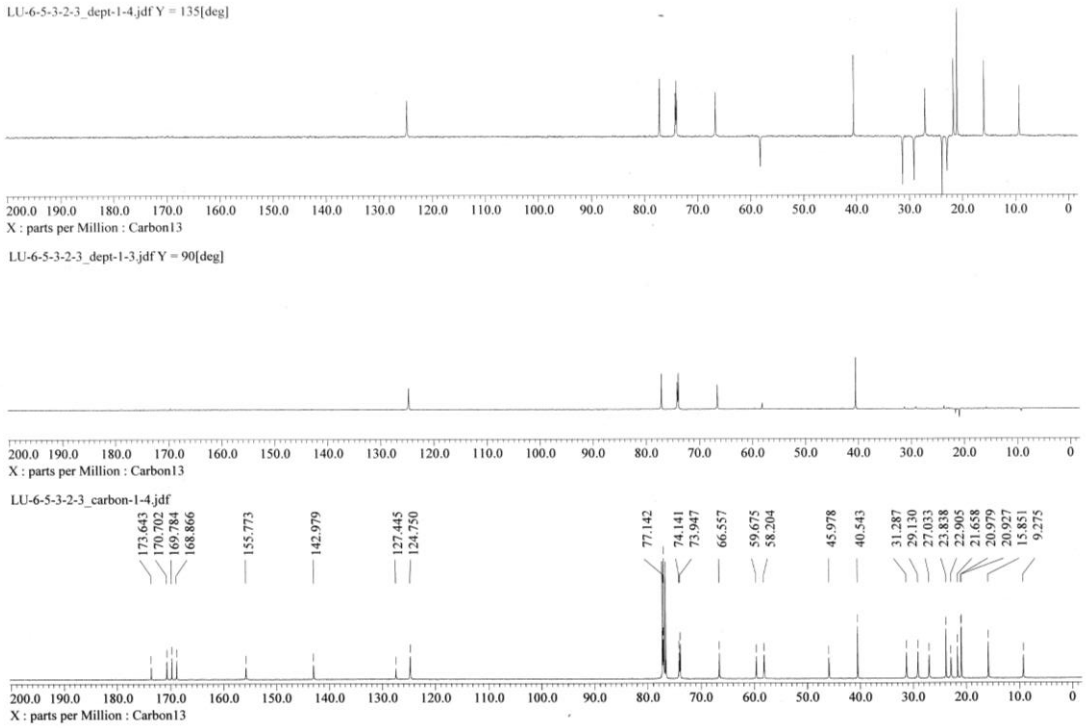

S5. DEPT spectrum of compound **1** in $CDCl_3$

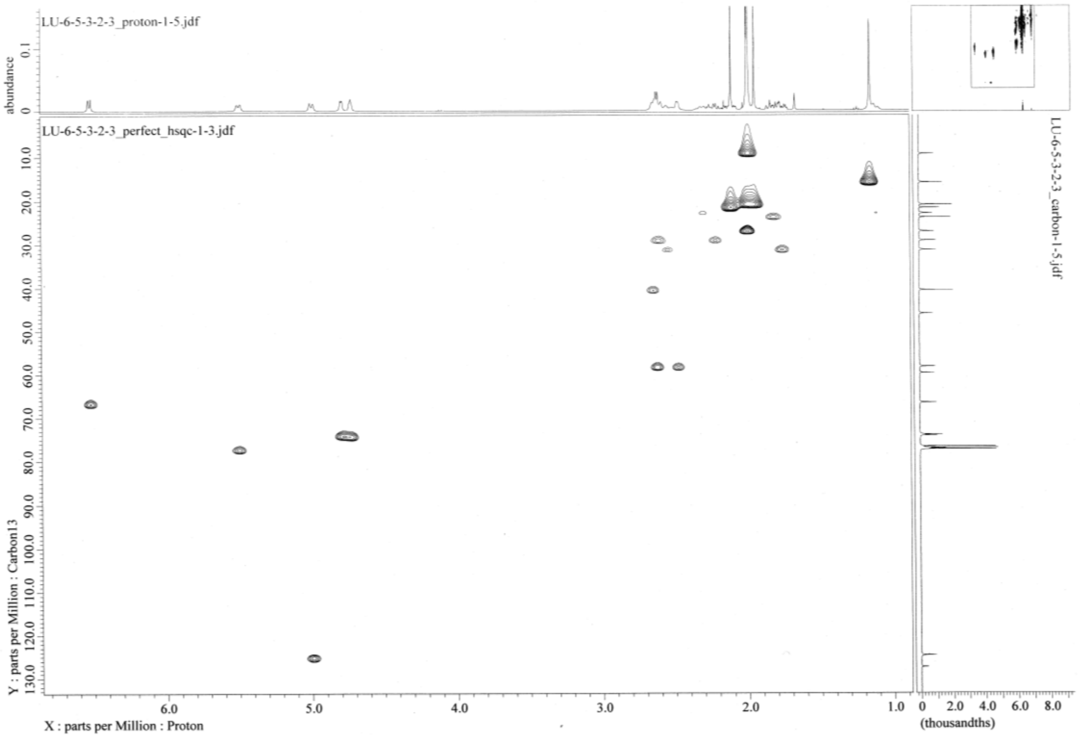

S6. HSQC spectrum of compound **1** in $CDCl_3$

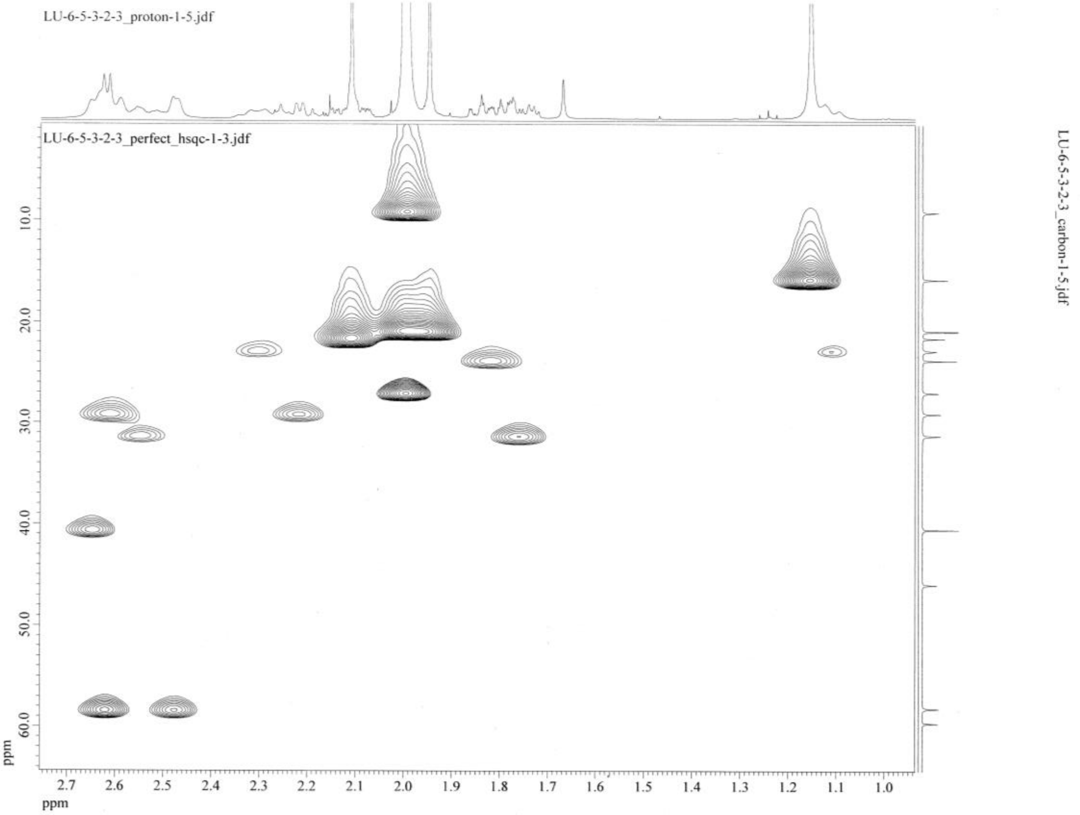

S7. HSQC spectrum of compound **1** in $CDCl_3$

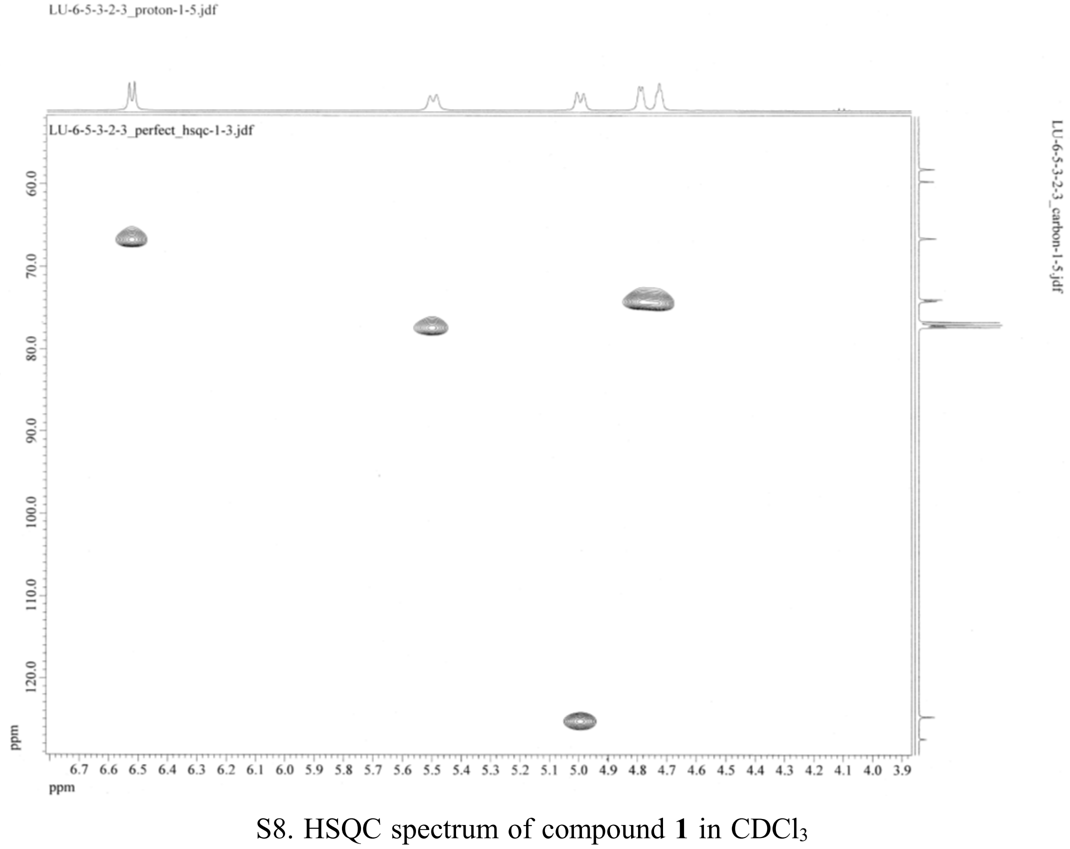

S8. HSQC spectrum of compound **1** in $CDCl_3$

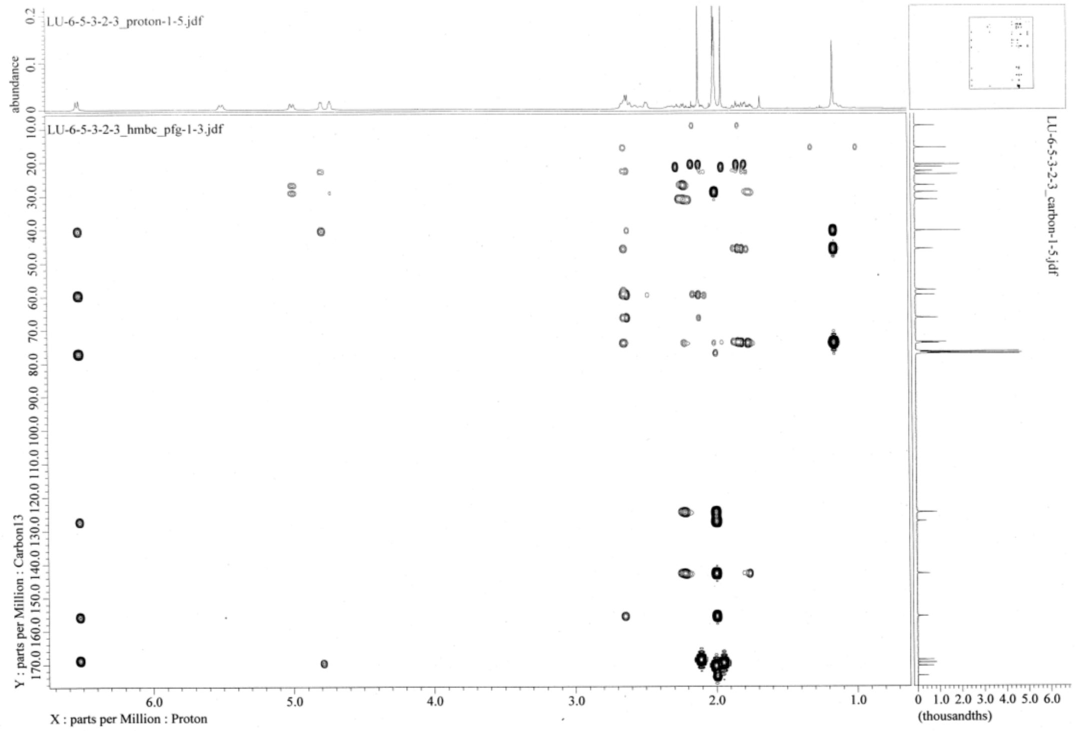

S9. HMBC spectrum of compound **1** in $CDCl_3$

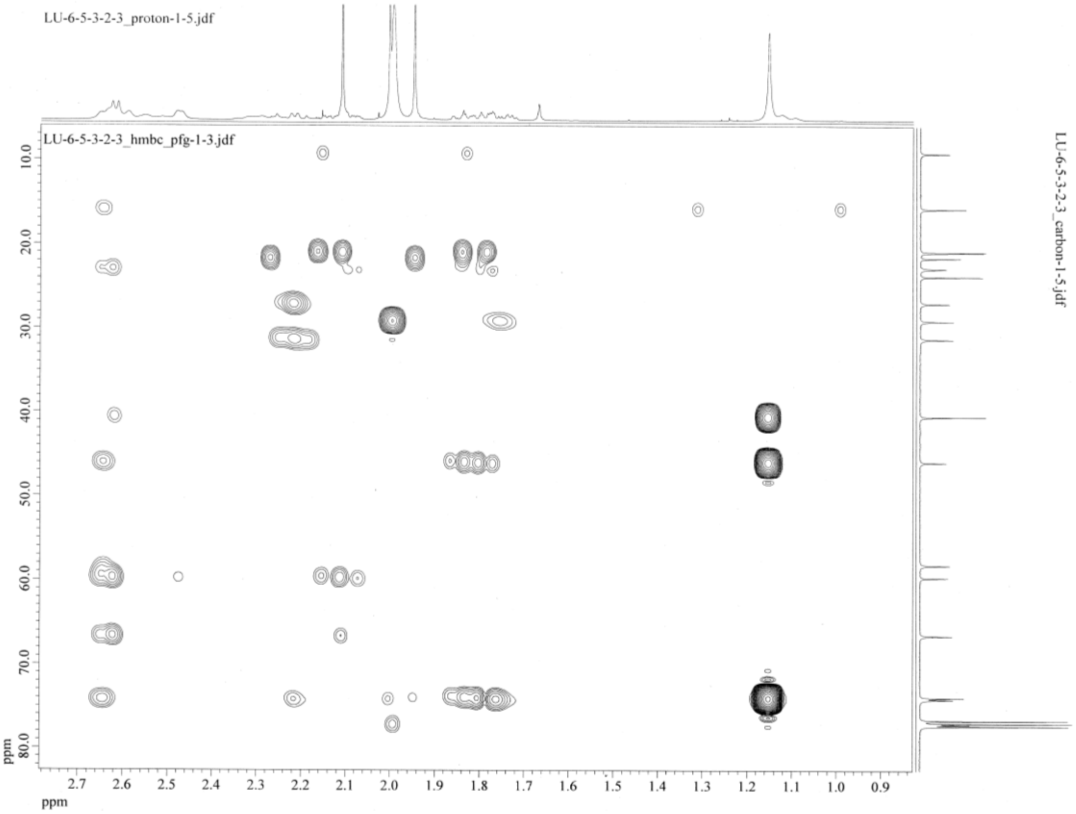

S10. HMBC spectrum of compound **1** in $CDCl_3$

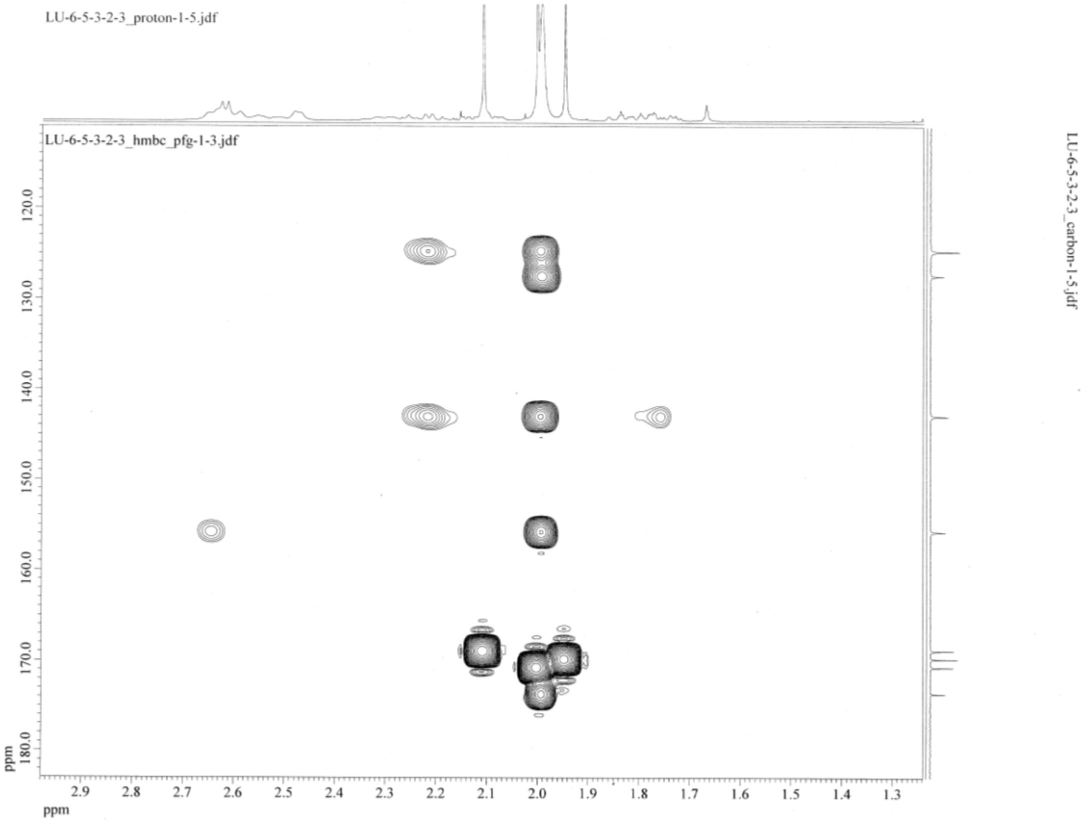

S11. HMBC spectrum of compound **1** in $CDCl_3$

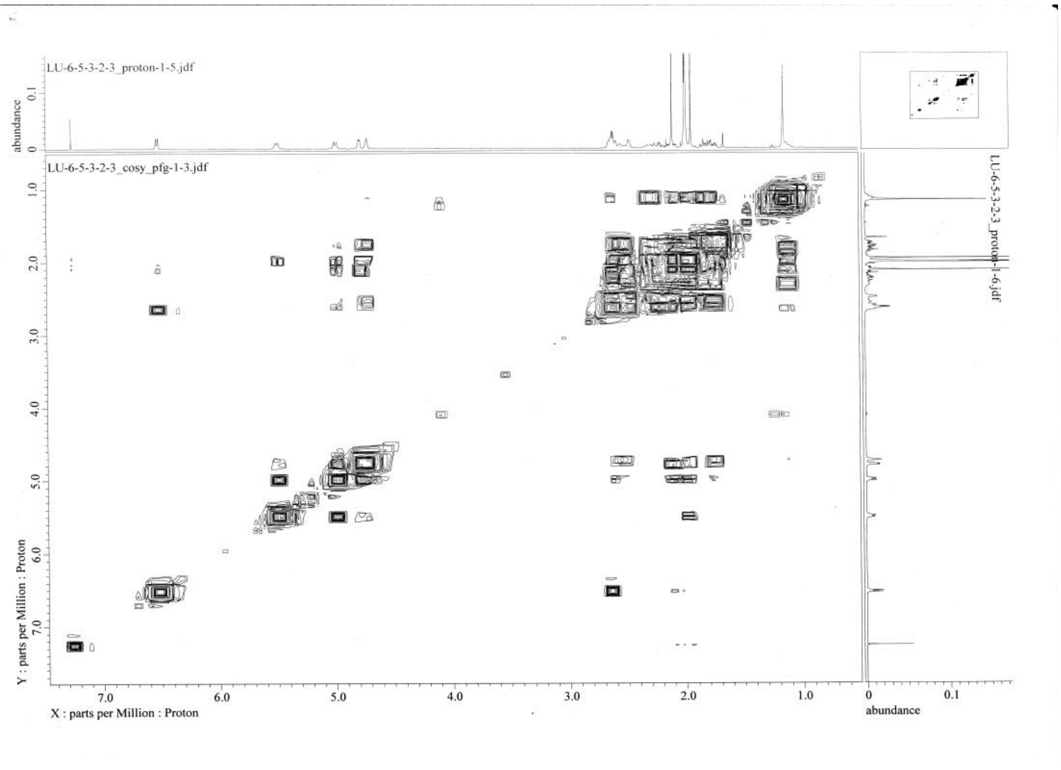

S12. ^{1}H-^{1}H COSY spectrum of compound **1** in $CDCl_3$

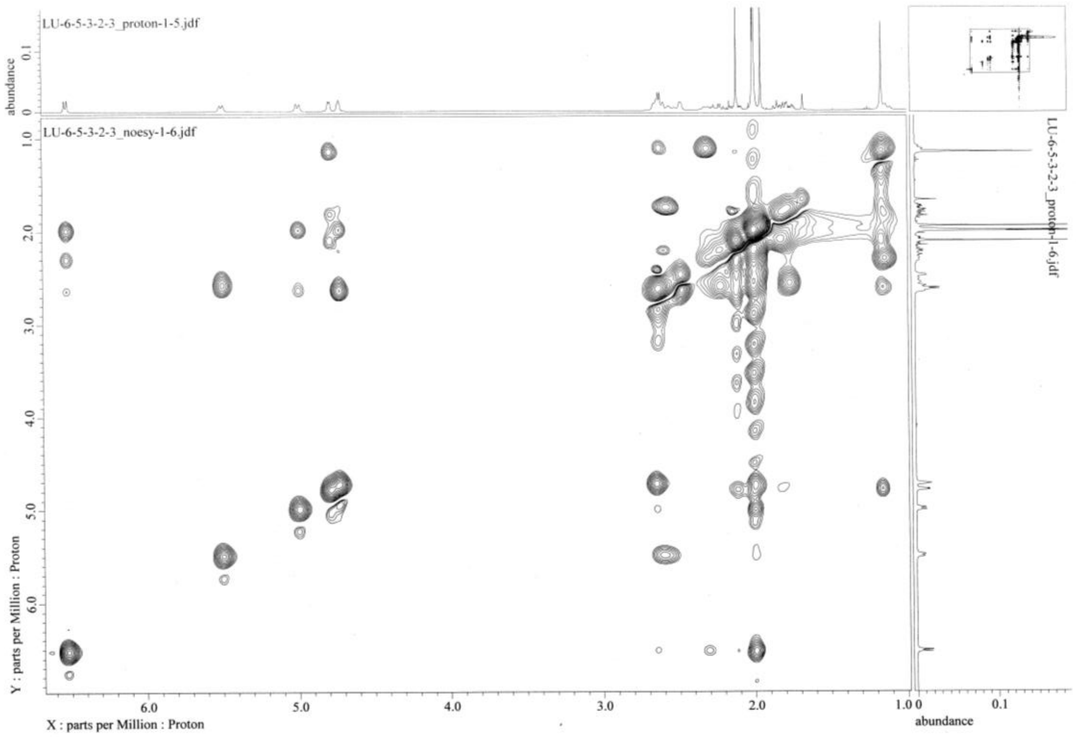

S13. NOESY spectrum of compound **1** in $CDCl_3$

S14. Single-crystal X-ray Crystallography of compound **1**

Table 1. Crystal data and structure refinement for ic20114.

Identification code	ic20114	
Empirical formula	C26 H35 O9.50	
Formula weight	499.54	
Temperature	200(2) K	
Wavelength	1.54178 Å	
Crystal system	Orthorhombic	
Space group	$P2_12_12_1$	
Unit cell dimensions	a = 9.8842(2) Å	α= 90°.
	b = 15.5702(2) Å	β= 90°.
	c = 17.0502(3) Å	γ = 90°.
Volume	2624.01(8) $Å^3$	
Z	4	
Density (calculated)	1.264 Mg/m^3	
Absorption coefficient	0.800 mm^{-1}	
F(000)	1068	
Crystal size	0.255 x 0.233 x 0.114 mm^3	
Theta range for data collection	3.844 to 74.977°.	
Index ranges	-12<=h<=12, -14<=k<=19, -19<=l<=21	
Reflections collected	12468	
Independent reflections	5385 [R(int) = 0.0341]	
Completeness to theta = 67.679°	99.6 %	
Absorption correction	Semi-empirical from equivalents	
Max. and min. transmission	0.7539 and 0.6703	
Refinement method	Full-matrix least-squares on F^2	
Data / restraints / parameters	5385 / 1 / 335	
Goodness-of-fit on F^2	1.062	
Final R indices [I>2sigma(I)]	R1 = 0.0396, wR2 = 0.1090	
R indices (all data)	R1 = 0.0404, wR2 = 0.1101	
Absolute structure parameter	0.07(5)	
Extinction coefficient	n/a	
Largest diff. peak and hole	0.235 and -0.308 e.$Å^{-3}$	

Table 2. Atomic coordinates (x 10^4) and equivalent isotropic displacement parameters ($Å^2$x 10^3) for ic20114. U(eq) is defined as one third of the trace of the orthogonalized U^{ij} tensor.

	x	y	z	U(eq)
O(1)	10355(2)	6980(1)	7684(1)	36(1)
O(2)	10239(3)	5597(2)	7317(2)	60(1)
O(2')	10498(9)	5569(4)	7837(7)	60(1)
O(3)	4211(2)	6663(1)	9541(1)	38(1)
O(4)	2022(2)	7041(1)	9536(1)	46(1)
O(5)	6755(2)	8663(1)	8680(1)	32(1)
O(6)	5357(2)	9796(1)	8653(2)	72(1)
O(7)	4561(2)	7853(1)	6728(1)	46(1)
O(8)	8233(2)	6859(1)	6265(1)	37(1)
O(9)	10148(2)	6671(1)	5578(1)	62(1)
C(1)	8303(2)	7777(1)	7415(1)	28(1)
C(2)	8910(2)	7000(1)	7859(1)	28(1)
C(3)	8720(2)	7020(1)	8753(1)	31(1)
C(4)	8328(2)	6146(1)	9105(1)	37(1)
C(5)	7109(2)	5744(1)	8716(1)	36(1)
C(6)	5884(2)	6099(1)	8695(1)	34(1)
C(7)	5431(2)	6901(1)	9114(1)	32(1)
C(8)	4971(2)	7653(1)	8610(1)	29(1)
C(9)	5901(2)	8210(1)	8126(1)	28(1)
C(10)	6710(2)	7697(1)	7489(1)	27(1)
C(11)	6020(2)	7901(1)	6704(1)	35(1)
C(12)	6628(3)	8646(2)	6265(1)	44(1)
C(13)	8075(3)	8407(2)	6025(1)	45(1)
C(14)	8694(2)	7697(1)	6540(1)	35(1)
C(15)	8963(2)	8622(1)	7692(1)	37(1)
C(16)	7353(3)	4900(2)	8303(2)	54(1)
C(17)	3653(2)	7787(1)	8720(1)	32(1)
C(18)	2713(2)	8441(2)	8382(2)	43(1)
C(19)	3159(2)	7147(1)	9293(1)	36(1)
C(20)	5313(2)	7224(2)	6276(1)	45(1)
C(21)	10905(3)	6238(2)	7472(2)	51(1)
C(22)	12376(3)	6327(2)	7287(2)	69(1)
C(23)	6353(2)	9455(1)	8904(1)	40(1)

C(24)	7320(4)	9839(2)	9474(2)	57(1)
C(25)	9066(3)	6417(2)	5794(1)	43(1)
C(26)	8475(4)	5557(2)	5597(2)	66(1)
O(10)	12212(9)	7987(6)	5649(5)	123(3)

Table 3. Bond lengths [Å] and angles [°] for ic20114.

O(1)-C(21)	1.327(3)
O(1)-C(2)	1.459(2)
O(2)-C(21)	1.225(4)
O(2')-C(21)	1.277(8)
O(3)-C(19)	1.352(3)
O(3)-C(7)	1.457(2)
O(4)-C(19)	1.209(3)
O(5)-C(23)	1.351(2)
O(5)-C(9)	1.450(2)
O(6)-C(23)	1.198(3)
O(7)-C(11)	1.445(3)
O(7)-C(20)	1.451(3)
O(8)-C(25)	1.340(3)
O(8)-C(14)	1.459(2)
O(9)-C(25)	1.199(3)
C(1)-C(15)	1.542(2)
C(1)-C(14)	1.547(3)
C(1)-C(2)	1.548(3)
C(1)-C(10)	1.584(3)
C(2)-C(3)	1.535(3)
C(3)-C(4)	1.538(3)
C(4)-C(5)	1.512(3)
C(5)-C(6)	1.331(3)
C(5)-C(16)	1.510(3)
C(6)-C(7)	1.507(3)
C(7)-C(8)	1.521(3)
C(8)-C(17)	1.333(3)
C(8)-C(9)	1.509(3)
C(9)-C(10)	1.568(2)
C(10)-C(11)	1.534(3)
C(11)-C(20)	1.461(3)
C(11)-C(12)	1.505(3)
C(12)-C(13)	1.534(4)
C(13)-C(14)	1.539(3)
C(17)-C(19)	1.479(3)
C(17)-C(18)	1.493(3)

C(21)-C(22)	1.494(4)
C(23)-C(24)	1.488(4)
C(25)-C(26)	1.499(4)
C(21)-O(1)-C(2)	118.43(18)
C(19)-O(3)-C(7)	109.79(16)
C(23)-O(5)-C(9)	117.27(17)
C(11)-O(7)-C(20)	60.57(15)
C(25)-O(8)-C(14)	117.35(18)
C(15)-C(1)-C(14)	104.96(16)
C(15)-C(1)-C(2)	110.67(16)
C(14)-C(1)-C(2)	108.21(15)
C(15)-C(1)-C(10)	117.57(16)
C(14)-C(1)-C(10)	108.55(16)
C(2)-C(1)-C(10)	106.58(15)
O(1)-C(2)-C(3)	108.86(15)
O(1)-C(2)-C(1)	107.28(15)
C(3)-C(2)-C(1)	115.01(15)
C(2)-C(3)-C(4)	113.69(16)
C(5)-C(4)-C(3)	113.28(16)
C(6)-C(5)-C(16)	119.7(2)
C(6)-C(5)-C(4)	124.36(19)
C(16)-C(5)-C(4)	115.9(2)
C(5)-C(6)-C(7)	127.0(2)
O(3)-C(7)-C(6)	105.74(16)
O(3)-C(7)-C(8)	103.33(16)
C(6)-C(7)-C(8)	117.36(16)
C(17)-C(8)-C(9)	125.55(18)
C(17)-C(8)-C(7)	109.52(18)
C(9)-C(8)-C(7)	124.68(17)
O(5)-C(9)-C(8)	106.14(15)
O(5)-C(9)-C(10)	113.72(15)
C(8)-C(9)-C(10)	113.41(15)
C(11)-C(10)-C(9)	105.80(15)
C(11)-C(10)-C(1)	110.88(16)
C(9)-C(10)-C(1)	121.46(15)
O(7)-C(11)-C(20)	59.93(15)
O(7)-C(11)-C(12)	116.87(19)

C(20)-C(11)-C(12)	119.90(19)
O(7)-C(11)-C(10)	114.10(17)
C(20)-C(11)-C(10)	119.94(19)
C(12)-C(11)-C(10)	114.56(18)
C(11)-C(12)-C(13)	108.59(19)
C(12)-C(13)-C(14)	113.06(18)
O(8)-C(14)-C(13)	109.60(18)
O(8)-C(14)-C(1)	107.69(15)
C(13)-C(14)-C(1)	113.18(17)
C(8)-C(17)-C(19)	108.00(18)
C(8)-C(17)-C(18)	131.4(2)
C(19)-C(17)-C(18)	120.59(19)
O(4)-C(19)-O(3)	122.1(2)
O(4)-C(19)-C(17)	128.7(2)
O(3)-C(19)-C(17)	109.19(18)
O(7)-C(20)-C(11)	59.50(14)
O(2)-C(21)-O(1)	123.3(2)
O(2')-C(21)-O(1)	116.6(4)
O(2)-C(21)-C(22)	123.5(2)
O(2')-C(21)-C(22)	119.0(5)
O(1)-C(21)-C(22)	112.1(2)
O(6)-C(23)-O(5)	123.0(2)
O(6)-C(23)-C(24)	125.7(2)
O(5)-C(23)-C(24)	111.3(2)
O(9)-C(25)-O(8)	124.3(2)
O(9)-C(25)-C(26)	125.0(2)
O(8)-C(25)-C(26)	110.7(2)

Symmetry transformations used to generate equivalent atoms:

Table 4. Anisotropic displacement parameters (Å^{2}x 10^3) for ic20114. The anisotropic displacement factor exponent takes the form: $-2\pi^2[h^2 a^{*2}U^{11} + ... + 2 h k a^* b^* U^{12}]$

	U^{11}	U^{22}	U^{33}	U^{23}	U^{13}	U^{12}
O(1)	29(1)	34(1)	44(1)	-9(1)	2(1)	2(1)
O(2)	53(1)	33(1)	94(2)	-14(1)	11(2)	7(1)
O(2')	53(1)	33(1)	94(2)	-14(1)	11(2)	7(1)
O(3)	38(1)	47(1)	30(1)	6(1)	6(1)	0(1)
O(4)	37(1)	63(1)	39(1)	0(1)	11(1)	-2(1)
O(5)	37(1)	26(1)	34(1)	-5(1)	-4(1)	1(1)
O(6)	65(1)	37(1)	112(2)	-25(1)	-23(1)	16(1)
O(7)	33(1)	69(1)	37(1)	-3(1)	-6(1)	5(1)
O(8)	40(1)	38(1)	31(1)	-5(1)	6(1)	-4(1)
O(9)	61(1)	68(1)	58(1)	-15(1)	28(1)	-4(1)
C(1)	30(1)	24(1)	31(1)	-1(1)	2(1)	-3(1)
C(2)	28(1)	25(1)	32(1)	-2(1)	1(1)	0(1)
C(3)	31(1)	32(1)	30(1)	-2(1)	-3(1)	4(1)
C(4)	43(1)	34(1)	35(1)	5(1)	0(1)	10(1)
C(5)	49(1)	26(1)	35(1)	5(1)	9(1)	1(1)
C(6)	42(1)	30(1)	32(1)	2(1)	4(1)	-4(1)
C(7)	33(1)	35(1)	27(1)	0(1)	3(1)	0(1)
C(8)	34(1)	30(1)	24(1)	-5(1)	0(1)	1(1)
C(9)	30(1)	26(1)	28(1)	-2(1)	-2(1)	2(1)
C(10)	29(1)	25(1)	27(1)	-1(1)	1(1)	-1(1)
C(11)	33(1)	42(1)	29(1)	0(1)	-1(1)	2(1)
C(12)	53(1)	44(1)	35(1)	11(1)	-2(1)	6(1)
C(13)	55(1)	42(1)	38(1)	14(1)	9(1)	-2(1)
C(14)	38(1)	32(1)	35(1)	2(1)	9(1)	-4(1)
C(15)	36(1)	25(1)	51(1)	-4(1)	1(1)	-5(1)
C(16)	63(2)	30(1)	70(2)	-8(1)	19(1)	-3(1)
C(17)	34(1)	35(1)	28(1)	-6(1)	1(1)	0(1)
C(18)	34(1)	45(1)	50(1)	0(1)	0(1)	9(1)
C(19)	36(1)	44(1)	27(1)	-6(1)	4(1)	0(1)
C(20)	40(1)	63(1)	32(1)	-7(1)	-4(1)	-4(1)
C(21)	41(1)	39(1)	71(2)	-11(1)	8(1)	9(1)
C(22)	35(1)	75(2)	97(2)	-42(2)	7(1)	7(1)
C(23)	46(1)	27(1)	47(1)	-5(1)	3(1)	1(1)

C(24)	82(2)	36(1)	54(1)	-12(1)	-14(1)	-2(1)
C(25)	53(1)	48(1)	29(1)	-5(1)	8(1)	1(1)
C(26)	81(2)	56(2)	60(2)	-24(1)	10(2)	-6(2)
O(10)	113(6)	132(6)	123(6)	28(5)	-33(5)	-26(5)

Table 5. Hydrogen coordinates (x 10^4) and isotropic displacement parameters ($Å^2$x 10^3) for ic20114.

	x	y	z	U(eq)
H(2)	8488	6463	7651	34
H(3A)	8007	7443	8883	38
H(3B)	9572	7217	8999	38
H(4A)	9106	5749	9057	45
H(4B)	8136	6221	9671	45
H(6)	5224	5816	8384	41
H(7)	6146	7094	9490	38
H(9)	5331	8645	7849	33
H(10)	6523	7076	7594	32
H(12A)	6639	9163	6603	53
H(12B)	6080	8774	5793	53
H(13A)	8652	8926	6057	54
H(13B)	8071	8213	5472	54
H(14)	9701	7723	6493	42
H(15A)	9015	8626	8265	56
H(15B)	8416	9110	7513	56
H(15C)	9876	8667	7471	56
H(16A)	6508	4700	8065	82
H(16B)	7672	4473	8683	82
H(16C)	8038	4979	7894	82
H(18A)	3141	9008	8400	65
H(18B)	1874	8452	8688	65
H(18C)	2507	8291	7837	65
H(20A)	5278	7266	5697	54
H(20B)	5368	6631	6484	54
H(22A)	12830	5775	7373	104
H(22B)	12778	6764	7628	104
H(22C)	12483	6499	6737	104
H(24A)	7017	10418	9616	86
H(24B)	8221	9870	9236	86
H(24C)	7359	9481	9947	86
H(26A)	8784	5380	5075	98

H(26B)	7486	5595	5601	98
H(26C)	8770	5133	5986	98
H(10A)	11648	7603	5783	184
H(10B)	12836	7960	5999	184

Table 6. Hydrogen bonds for ic20114 [Å and °].

D-H...A	d(D-H)	d(H...A)	d(D...A)	<(DHA)
O(10)-H(10A)...O(9)	0.85	2.10	2.895(9)	154.8
O(10)-H(10B)...O(7)#1	0.86	2.12	2.969(8)	171.9

Symmetry transformations used to generate equivalent atoms:
#1 x+1,y,z

Mass Spectrum SmartFormula Report

Analysis Info

Analysis Name	D:\Data\a2\LU745412_000046.d	2/17/2020 3:30:49 PM
Method	broadband first signal	Operator: YU HSIAO-CHING
Sample Name	LU-7-4-5-4-1-2	Instrument: BRUKER FT-MS solariX
Comment	ESI Positive	

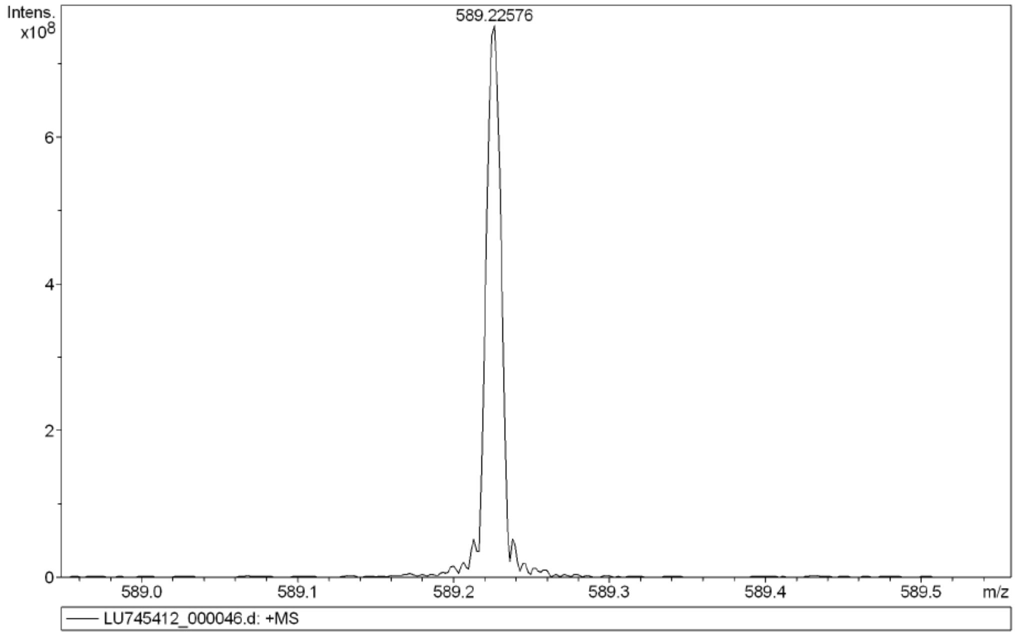

Meas. m/z	#	Formula	Score	m/z	err [mDa]	err [ppm]	mSigma	rdb	e⁻ Conf	N-Rule
589.22576	1	C 28 H 38 Na O 12	100.00	589.22555	-0.22	-0.37	10.4	9.5	even	ok

S15. HRESIMS spectrum of compound **2**

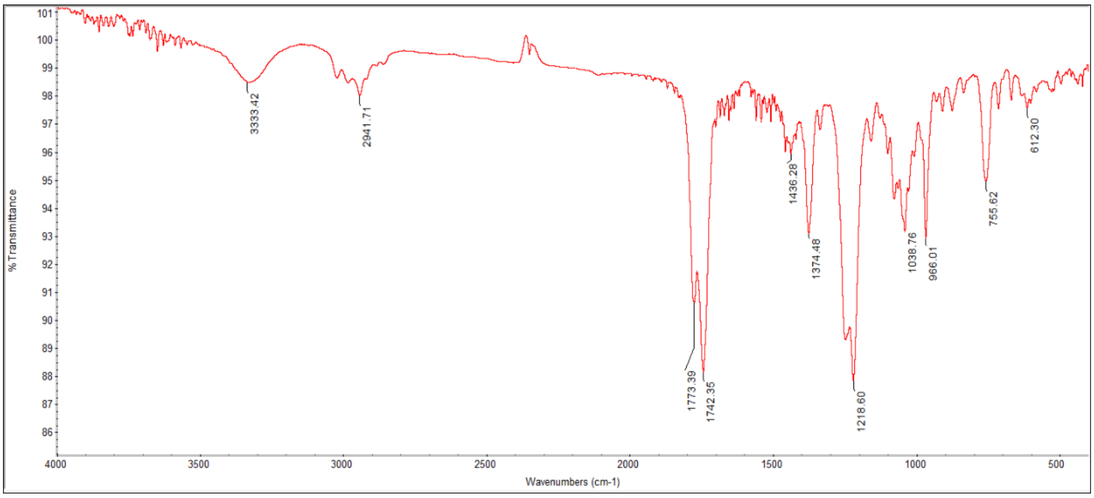

S16. IR spectrum of compound **2**

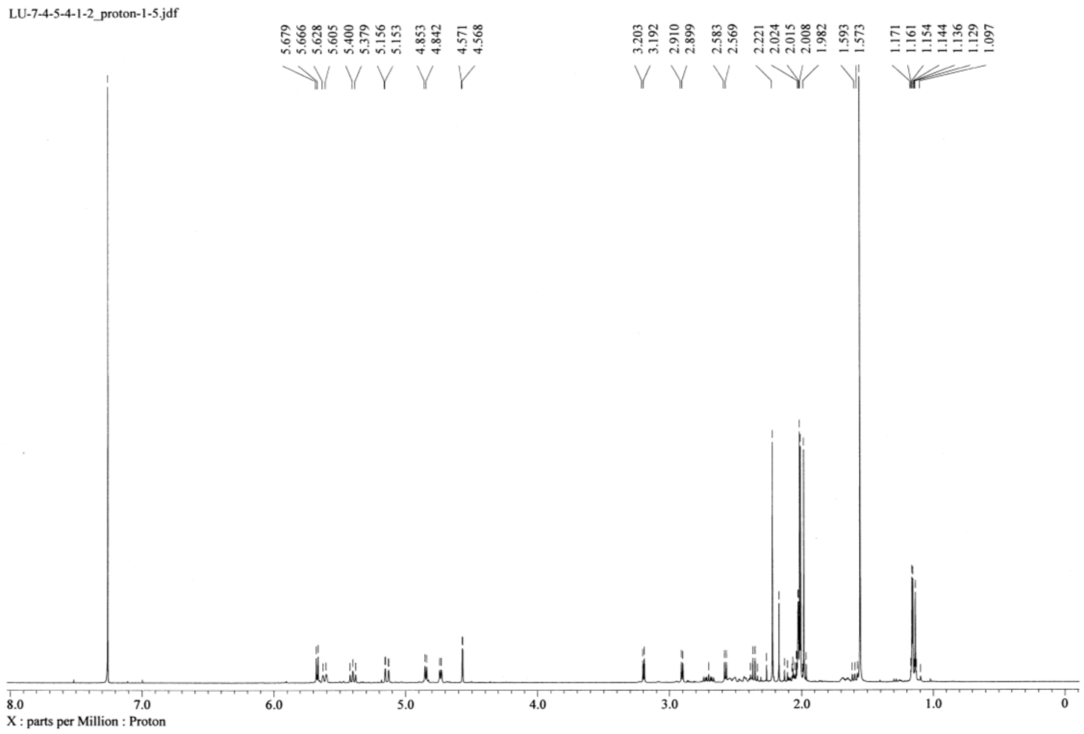

S17. ^{1}H NMR spectrum (400 MHz) of compound **2** in $CDCl_3$

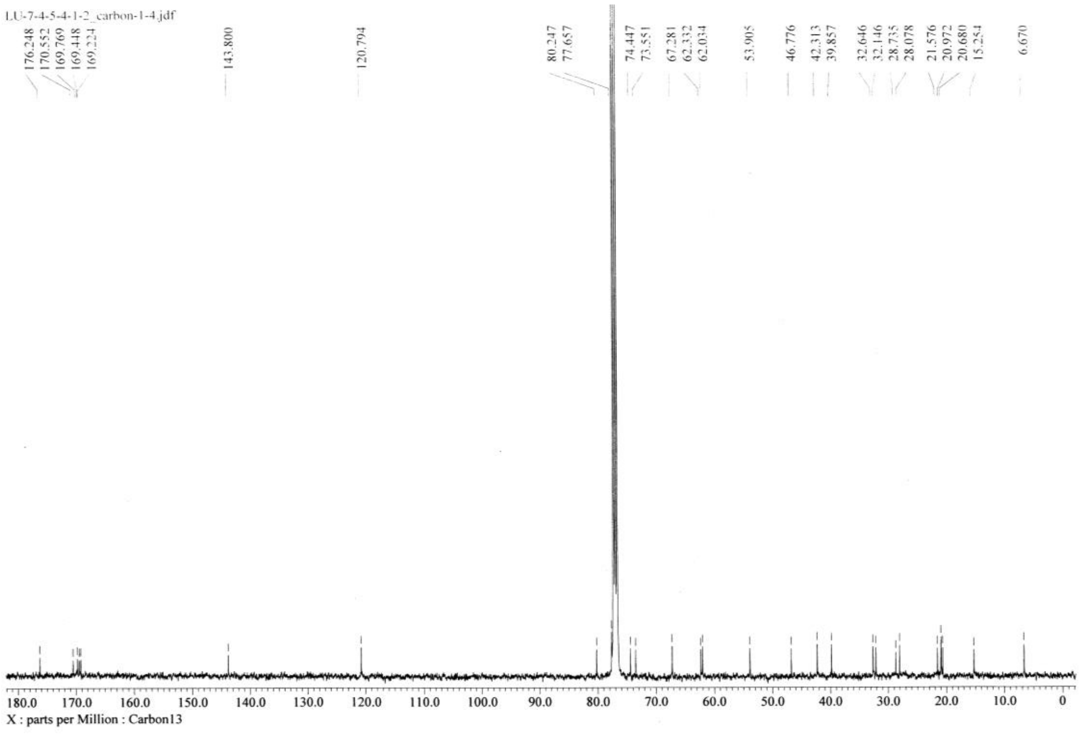

S18. ^{13}C NMR spectrum (100 MHz) of compound **2** in $CDCl_3$

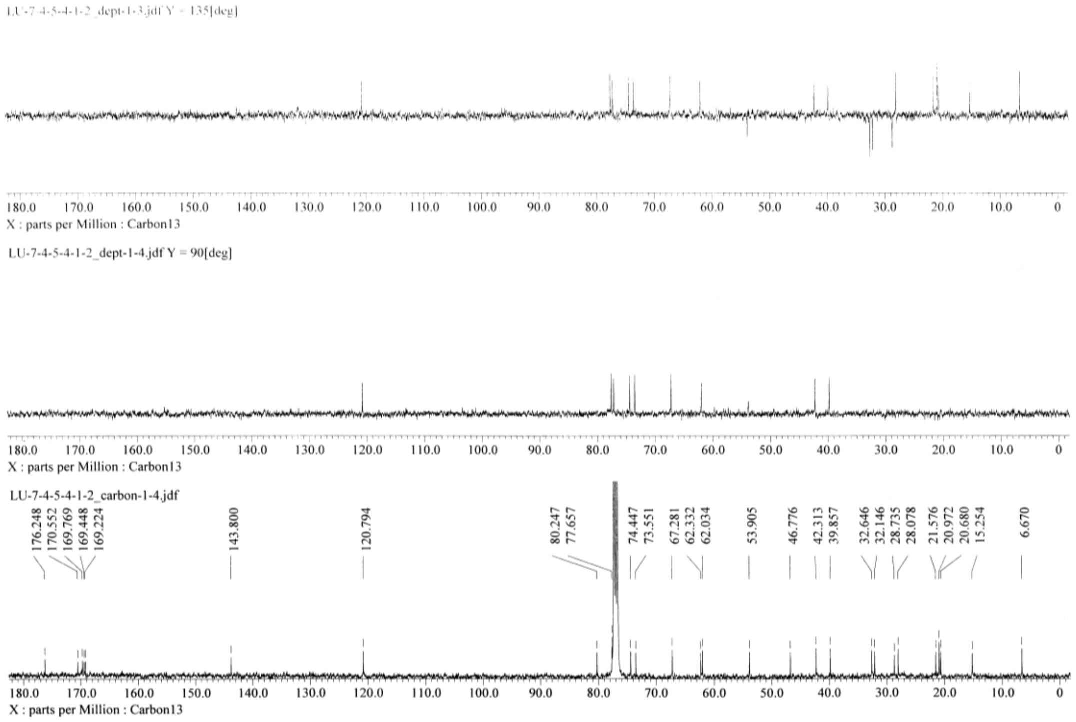

S19. DEPT spectrum of compound **2** in $CDCl_3$

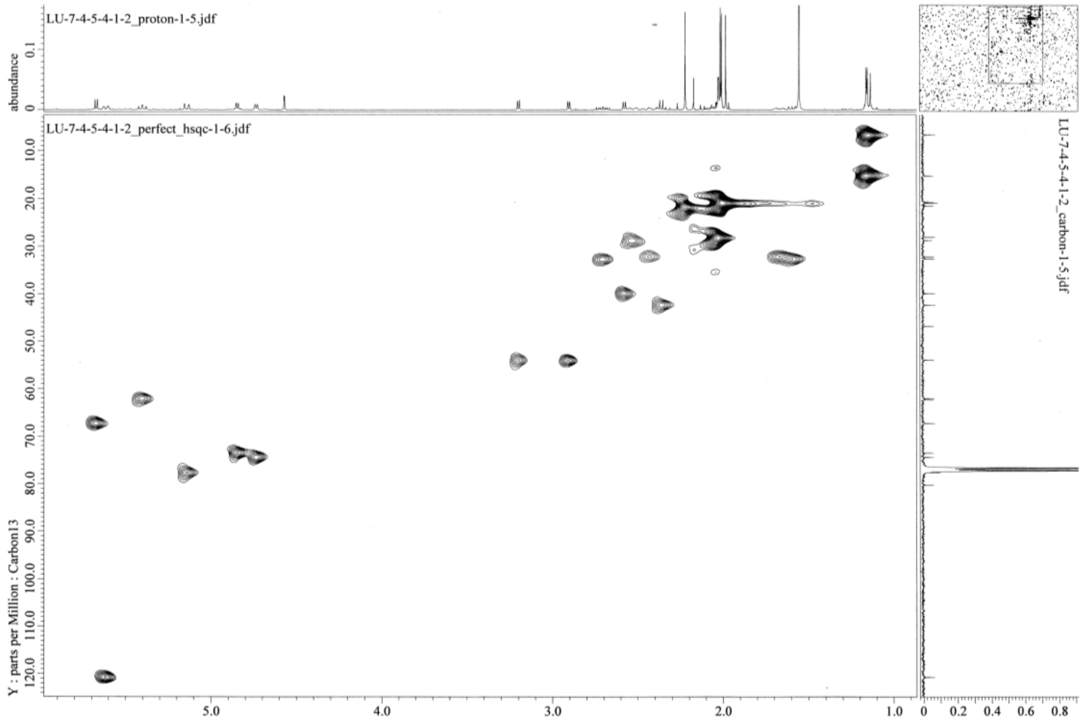

S20. HSQC spectrum of compound **2** in $CDCl_3$

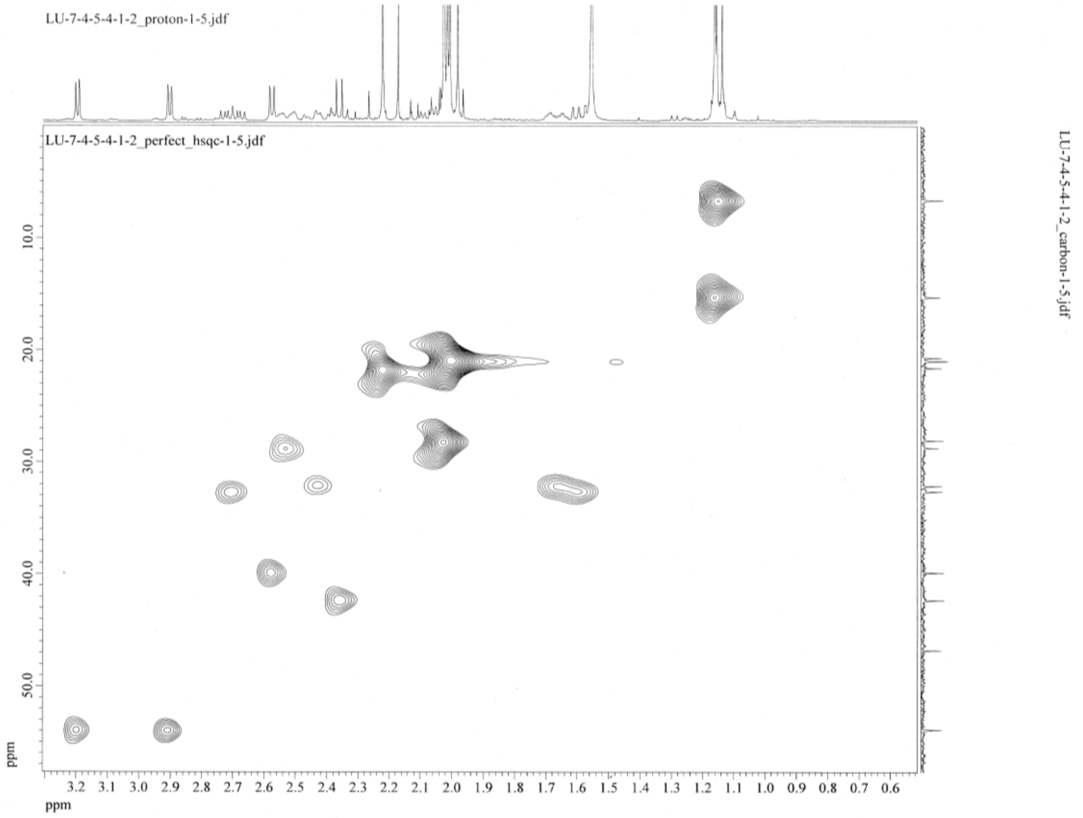

S21. HSQC spectrum of compound **2** in $CDCl_3$

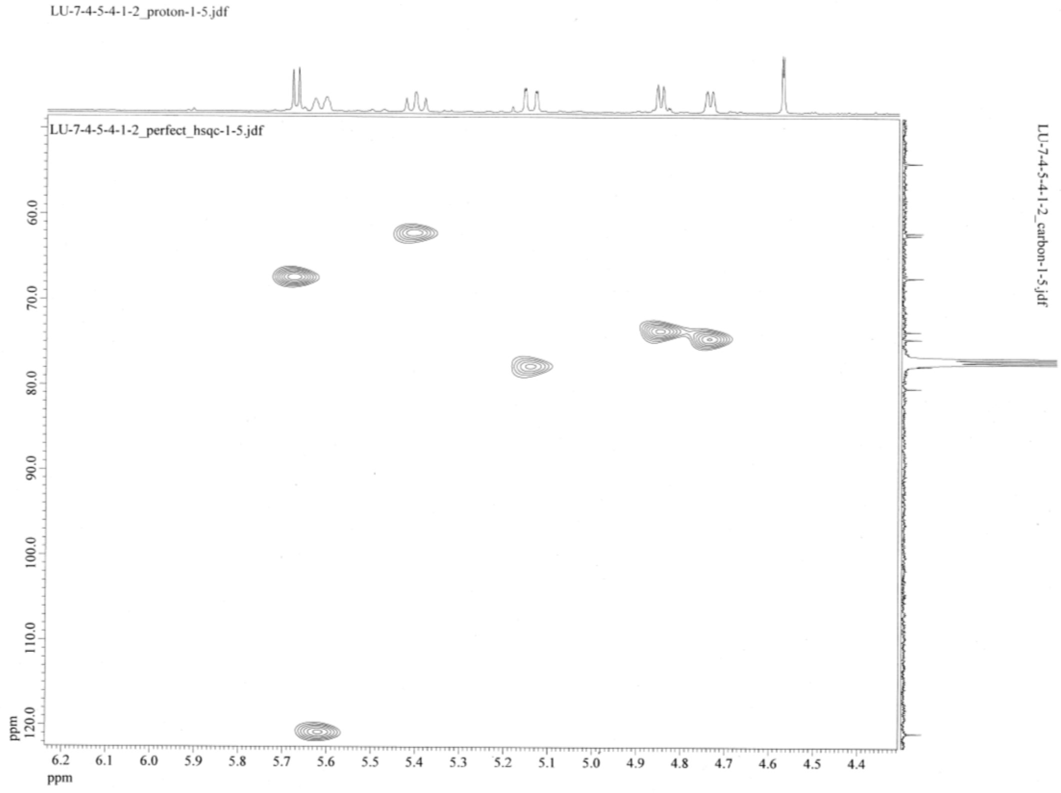

S22. HSQC spectrum of compound **2** in $CDCl_3$

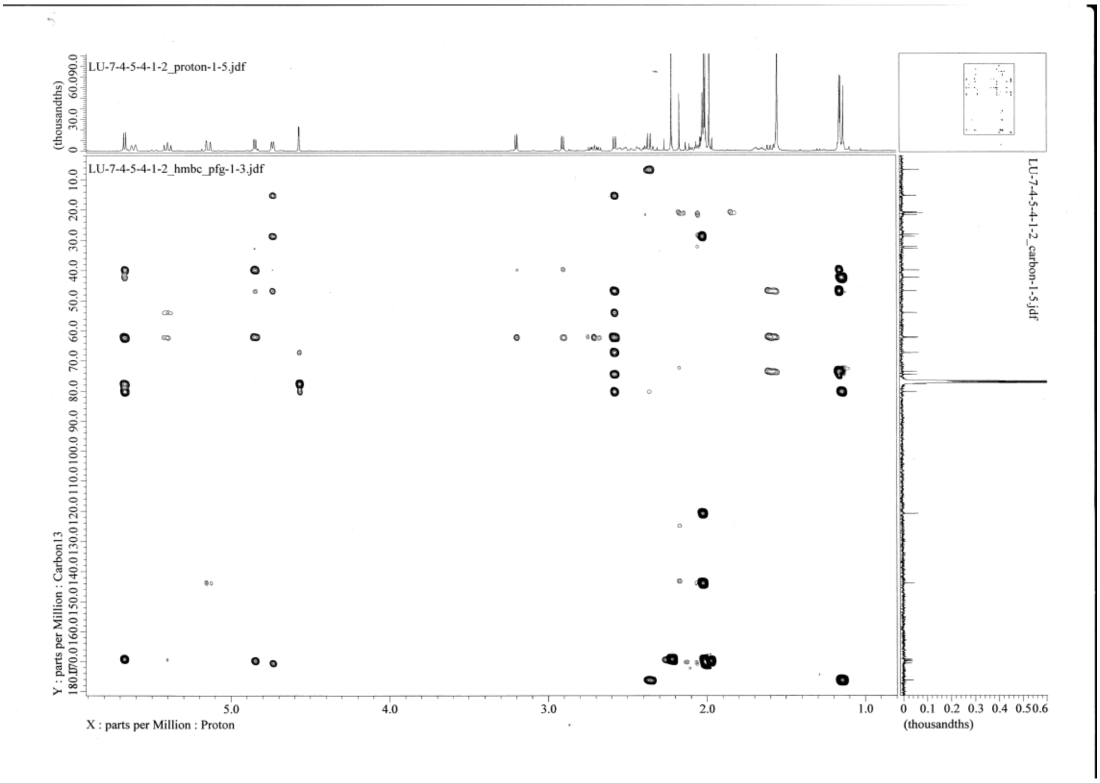

S23. HMBC spectrum of compound **2** in $CDCl_3$

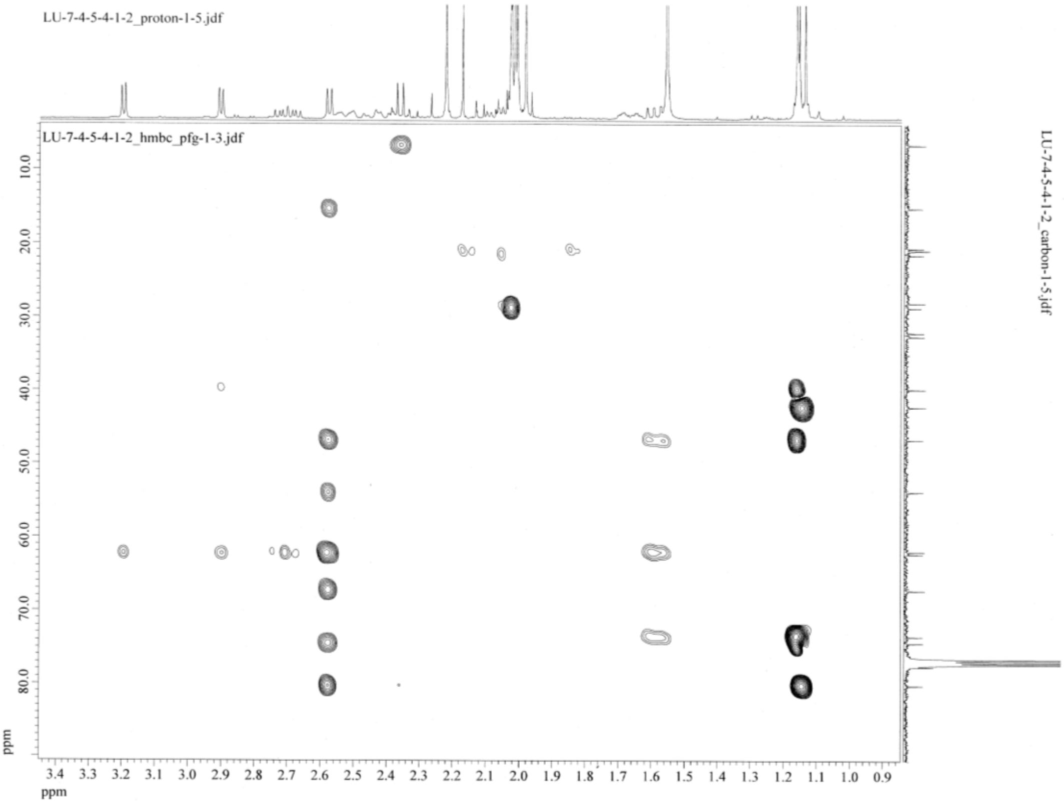

S24. HMBC spectrum of compound **2** in $CDCl_3$

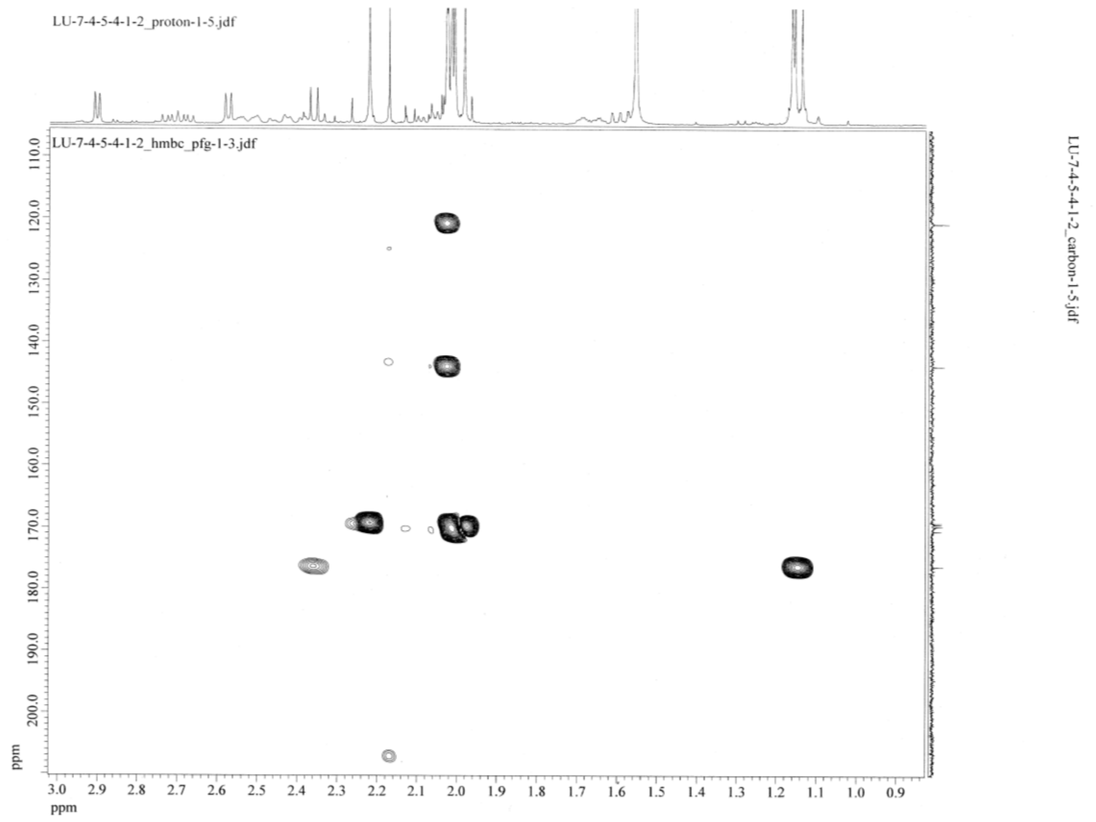

S25. HMBC spectrum of compound **2** in $CDCl_3$

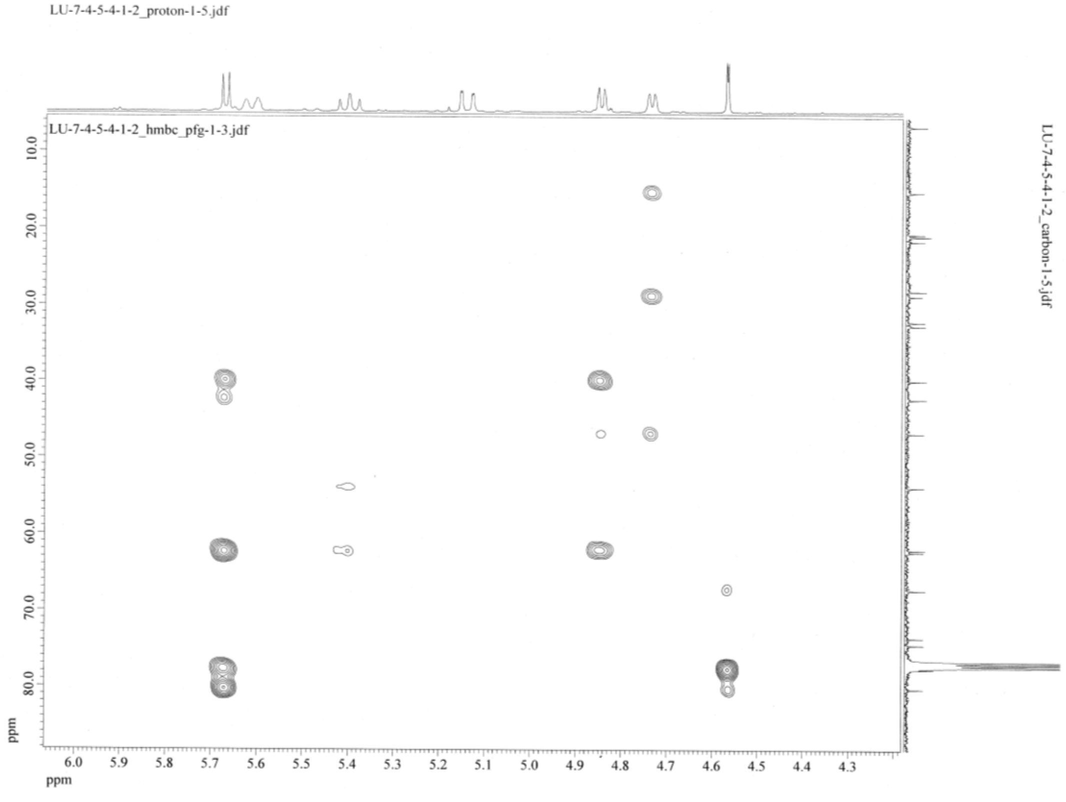

S26. HMBC spectrum of compound **2** in $CDCl_3$

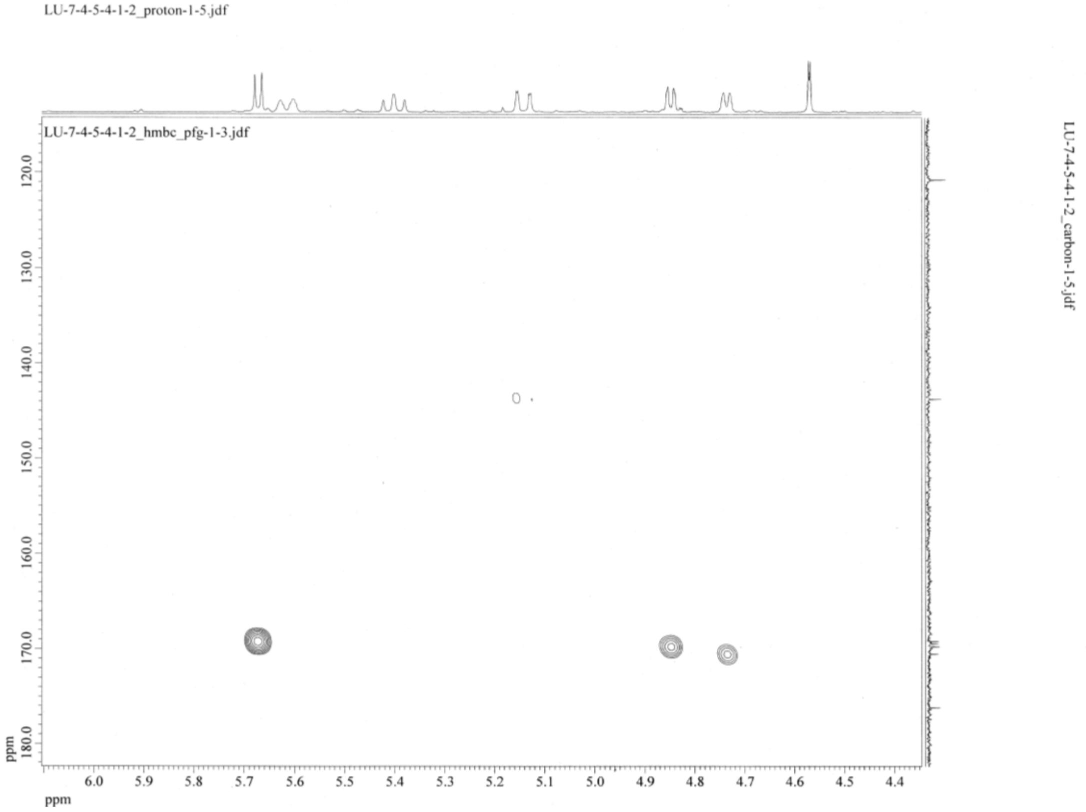

S27. HMBC spectrum of compound **2** in $CDCl_3$

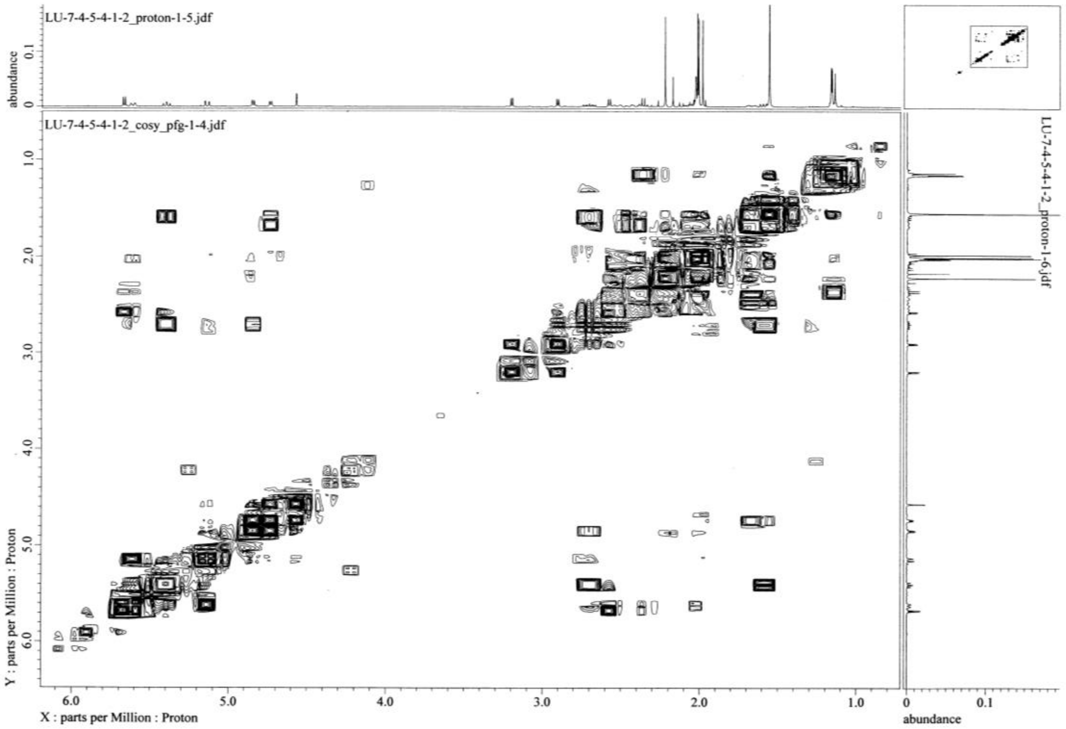

S28. ^1H-^1H COSY spectrum of compound **2** in $CDCl_3$

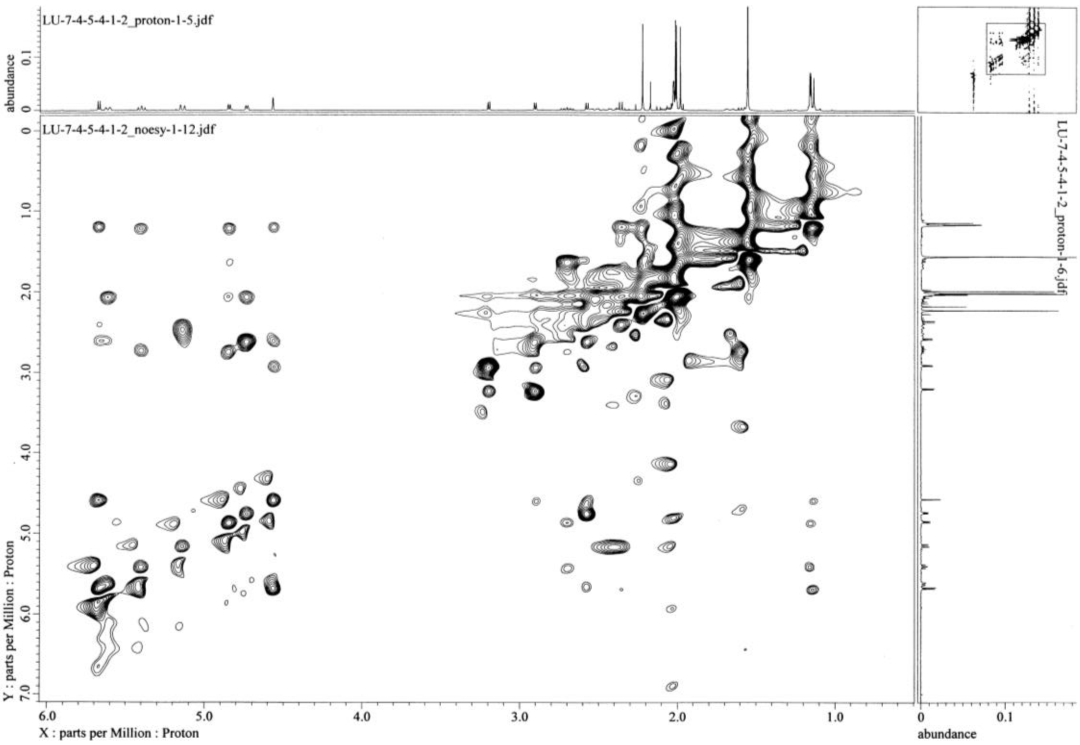

S29. NOESY spectrum of compound **2** in $CDCl_3$
